# Photochemical Pathways and Light-Enhanced Radical Scavenging Activity of 1,8-Dihydroxynaphthalene Allomelanin

**DOI:** 10.1021/jacs.5c01855

**Published:** 2025-03-07

**Authors:** Vasilis Petropoulos, Dario Mordini, Francesco Montorsi, Mert Akturk, Arianna Menichetti, Andrea Olivati, Annamaria Petrozza, Vittorio Morandi, Margherita Maiuri, Nathan C. Gianneschi, Marco Garavelli, Luca Valgimigli, Giulio Cerullo, Marco Montalti

**Affiliations:** † Dipartimento di Fisica, Politecnico di Milano, Piazza Leonardo da Vinci 32, Milano 20133, Italy; ‡ Department of Chemistry “Giacomo Ciamician”, University of Bologna,Via Selmi 2, Bologna 40126, Italy; § Dipartimento di Chimica industriale “Toso Montanari”, Università di Bologna, via Piero Gobetti 85, Bologna 40129, Italy; ∥ Center for Nano Science and Technology @PoliMi, Istituto Italiano di Tecnologia, Via Rubattino 81, Milan 20134, Italy; ⊥ Istituto per la Microelettronica e i Microsistemi (IMM), Consiglio Nazionale delle Ricerche (CNR), via Gobetti 101, Bologna 40129, Italy; # Departments of Chemistry, Materials Science & Engineering, Biomedical Engineering and Pharmacology, Northwestern University, Evanston, Illinois 60208, United States; ¶ Department of Chemistry &Biochemistry, University of California San Diego, La Jolla, California 92093, United States

## Abstract

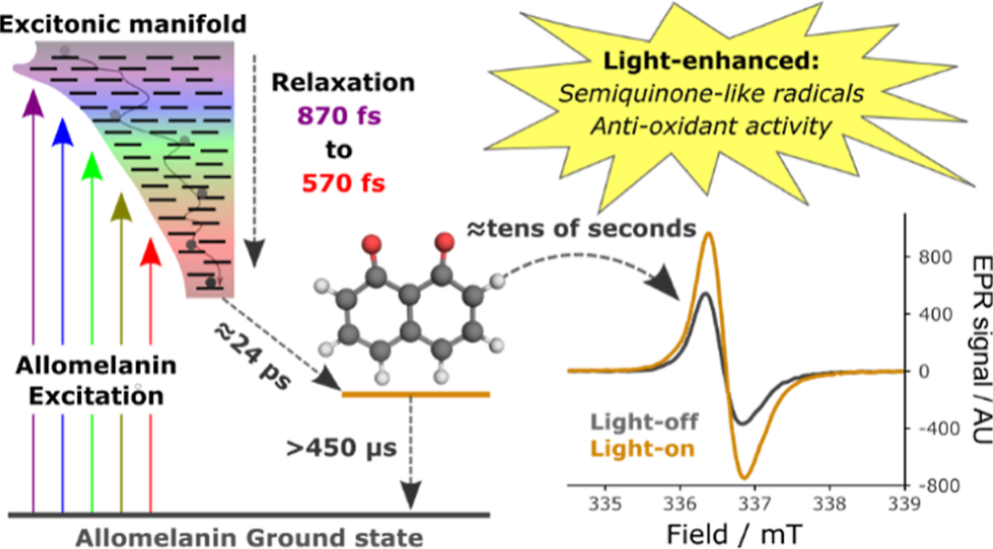

Melanins play important roles in nature, particularly in coloration and photoprotection, where interaction with light is essential. Biomimetic melanins represent an advantageous alternative to natural melanin for technological applications, sharing the same unique biocompatibility, as well as optoelectronic properties. Allomelanin, derived from 1,8-dihydroxynaphthalene, has been reported to exhibit even better photoprotective and antioxidant properties than the most studied example of biomimetic melanin, polydopamine. However, the interaction of allomelanin with light remains largely unexplored. Here we report the excited state dynamics of allomelanin in a wide range of time windows from femtoseconds to microseconds to minutes, using different experimental techniques, i.e., ultrafast transient absorption, nanosecond transient absorption, X-band electron paramagnetic resonance and radical quenching assays. We find that the photophysics of allomelanin starkly differs from that of the widely studied polydopamine, with broadband excitonically coupled states funneling the absorbed energy to a lower energy species in less than 1 ps. Independent of the excitation wavelength, a long-lived (>450 μs) photoproduct is populated in ≈24 ps. Quantum chemistry calculations suggest that the photoproduct primarily exhibits the character of localized 1,8-naphthoquinone radical anions. This light-driven increase in the anionic semiquinone-like radical concentration enhances the antioxidant activity of allomelanin. These results suggest that the two mechanisms considered at the basis of photoprotection, light-extinction and antioxidant action, are indeed synergistic in allomelanin and not independent, paving the way for new applications of allomelanin in nanomedicine, photocatalysis, energy conversion and environmental remediation.

## Introduction

Melanins, a diverse family of pigments ubiquitous in nature, play pivotal roles in living species by providing coloration and protection against the detrimental effects of solar radiation.^1−3^ Their photoprotective function arises from two key mechanisms: (i) light absorption across a broad range of photon energies, from ultraviolet (UV) to near-infrared (NIR), followed by efficient nonradiative energy dissipation, and (ii) the ability to neutralize light-induced harmful radicals, a function linked to their intrinsic ground electronic state properties.^4,5^ Transient optical spectroscopy techniques have identified melanin derivatives as effective UV-to-NIR filters, exhibiting dominant nonradiative decay processes on sub-10 ps time scales.^6−10^ Electron paramagnetic resonance (EPR), on the other hand, has provided insights into the spin properties of melanins, revealing that a persistent, stable free radical is central to their antioxidant and radical scavenging activity.^5,11−14^ However, the two photoprotection mechanisms are typically treated as independent processes, highlighting the need for a clearer understanding of their possible synergistic link.

Formed through the oxidation/polymerization of molecular units, melanins are categorized based on their precursors and functionality.^3^ Biomimetic melanins, synthesized using various oxidants, with atmospheric oxygen being the simplest, are efficiently internalized by living species and possess optoelectronic properties similar to their natural counterparts.^15−17^ Among the different types of melanins, the nitrogenous eumelanin, found in the eyes, hair, and skin of living organisms, is the most thoroughly examined.^3^ On the other hand, allomelanin, derived from the Greek prefix “allo-” meaning “different”, belongs to a nitrogen-free category of melanins and is commonly found in fungi, bacteria, and plants.^3,18^ Allomelanin has been reported to exhibit superior properties compared to the archetypal eumelanin analogue, polydopamine (PDA),^19^ particularly in terms of radiation protection, including ionizing radiation, and antioxidant activity.^17,20−27^ Consequently, allomelanin represents a promising alternative to PDA in numerous applications of significant social and economic importance.^28^

Although the scientific literature frequently generalizes the optical properties observed in the extensively studied eumelanins to the broader melanin family,^29−33^ an in-depth investigation of photoinduced processes in other promising melanins, such as allomelanin, is still missing. Here we investigate the photophysical and photochemical behavior of allomelanin nanoparticles (NPs) derived from the 1,8-dihydroxynaphthalene (1,8-DHN) precursor. By combining optical transient absorption (TA) spectroscopy and X-band EPR, we explore a wide range of time scales ranging from femto- and micro-seconds to seconds. Additionally, with the aid of quantum chemistry calculations, we unify TA and EPR findings into a single mechanistic framework. Our results reveal that allomelanin comprises coupled electronic transitions, demonstrating a significant departure from the established transient spectral heterogeneity of eumelanins^7−10,30^ and disordered carbonaceous nanomaterials.^34−37^ When exposed to light tunable from UV to visible wavelengths, allomelanin efficiently channels the excitation to similarly distributed low-energy excited states on the subpicosecond time scale. Independent of the excitation wavelength, long-lived (>450 μs) anionic semiquinone-like radicals form within 24 ps through electron and proton transfer reactions between hydroquinone- and quinone-like pairs. Quantum chemistry calculations suggest that these radicals can be well described as localized 1,8-naphthoquinone radical anions, offering additional structural insight into the inherent disorder of allomelanin. Furthermore, exploiting X-band EPR spectroscopy and radical scavenging assays, we demonstrate that the localized semiquinone-like radicals persist for up to ∼40 s, enhancing allomelanin’s antioxidant activity under light exposure compared to dark conditions. As a result, the radical scavenging activity of allomelanin increases upon light absorption, demonstrating a synergy between the two mechanisms at the basis of photoprotection, light-extinction and antioxidant activity.

## Experimental Section

### Allomelanin NPs Synthesis

Allomelanin NPs were synthesized as reported by Zhou et al.^17^ In short, 20 mg of 1,8-DHN were sonicated in 1 mL of acetonitrile until the solute was completely dissolved. The solution was then transferred to a round-bottom flask containing 19 mL of ultrapure water under magnetic stirring. Next, 124.9 μL of 1 N NaIO_4_ were added to the mixture. After 12 h, the NPs were isolated from the solution by centrifugation (10,000 rpm, 10 min, 25 °C) and washed with ultrapure water five times.

### UV–Vis Absorption Spectra

The experiments were carried out in air-equilibrated solutions at 25 °C. UV–Vis absorption spectra were recorded using a PerkinElmer LAMBDA 650 spectrophotometer in the wavelength range of 190–800 nm, with quartz cells having a path length of 1.0 cm.

### Emission Spectroscopy

Excitation–emission maps of the sample were acquired using a FluoroMax-4 system (HORIBA Scientific; Kyoto, Japan), in the wavelength range of 250–800 nm for excitation and emission, using quartz cells with a path length of 1.0 cm. The fluorescence quantum yields (uncertainty, ±15%) for UV (<320 nm) and visible (500–600 nm) excitations were calculated according to standard methods using naphthalene in cyclohexane (ϕ = 23%)^38^ and rhodamine 101 in ethanol (ϕ = 100%)^39^ as references, respectively.

### Fourier Transform Infrared Spectroscopy

Fourier transform infrared (FTIR) spectra were recorded using a Bruker Alpha Platinum-ATR. Prior to measurements, the samples were stored overnight in a dryer connected to a vacuum pump. They were then ground into a fine powder using a jade mortar and placed into the sample holder of the instrument as is.

### Dynamic Light Scattering

Dynamic light scattering (DLS) measurements were performed with Zetasizer Nano ZS Malvern Panalytical using PMMA semimicro cuvettes (BRAND).

### Electron Paramagnetic Resonance

EPR spectra were recorded in X-band with cavity thermostated at 30 °C. UV irradiation in cavity was provided by a mercury-xenon lamp (240–400 nm, max 4500 mW/cm^2^) on samples of allomelanin in water (50 μL) in 1 mm ID Suprasil quartz tubes. Spectra were recorded with field center at 336.45 mT, with field sweep 8 mT, modulation frequency 100 kHz, modulation amplitude 0.1–0.2 mT, sweep time 60 s. Microwave power was set at 0.7 mW which corresponded to half-saturation (*P*_1/2_) determined in preliminary power sweep experiments. UV irradiation power ranged from 20% to 100% of the nominal lamp power. Measured *g*-factors were corrected with respect to those of 2,2,6,6-tetramethylpyperidine-*N*-oxyl (TEMPO) radical in water (*g* = 2.0062)^40^ and TEMPO radical in benzene (*g* = 2.0064).^41^

### Scanning Electron Microscopy

Scanning electron microscope (SEM) images were obtained using a ZEISS Leo 1530 microscope at a voltage of 5 kV with an In-lens detector. For the acquisition, allomelanin NPs in water were dried on a SEM stub and coated with gold.

### 2,2-Diphenyl-1-picrylhydrazyl Radical Scavenging Assay

2,2-Diphenyl-1-picrylhydrazyl (DPPH) radical scavenging activity was measured according to the literature.^42^ Briefly, 0.06 mM of DPPH solution in 95% ethanol was prepared before use, and then allomelanin NPs were dispersed in water and mixed with 3.0 mL of the DPPH solution. The scavenging activity was evaluated by monitoring the absorbance decrease at 517 nm after it remained in the dark for 30 min. DPPH radical scavenging activity was calculated as % deg = [1 – (*A*_*i*_ – *A*_*j*_)/*A*_c_] × 100%, where *A*_c_ is the absorbance of DPPH solution without allomelanin NPs, *A*_*i*_ is the absorbance of the samples of allomelanin NPs with DPPH solution, and *A*_*j*_ is the absorbance of the samples of allomelanin NPs themselves without DPPH solution.

### Transient Absorption Spectroscopy

For the femtosecond transient absorption (fs-TA) measurements an amplified Ti:sapphire laser (Coherent Libra, 800 nm, 80 fs pulse duration, 1 kHz repetition frequency) was utilized.^43^ The probe pulses were obtained by white-light continuum (WLC) generation focusing the 800 nm beam into a rotated 1 mm-thick CaF_2_ plate, resulting in a probe spectrum ranging from 320 to 680 nm. For detection in the NIR range, a portion of the 800 nm beam was used to pump a noncollinear optical parametric amplifier (NOPA) seeded by WLC generated in a 1 mm-thick sapphire plate, producing pulses centered at 1200 nm in the NIR. Subsequently, the NIR probe pulses were generated by WLC after focusing the output of the NIR NOPA into a YAG plate, resulting in a probe spectrum ranging from 580 to 950 nm. The 266 nm pump beam was obtained by third harmonic generation of the 800 nm pulse in a two-step process: (i) a fraction of the fundamental beam underwent frequency doubling through a β-barium borate crystal; (ii) the remaining fundamental beam and the second harmonic generated in the first crystal were combined in a second β-barium borate crystal for sum-frequency generation. The 400 nm pump beam was generated through second harmonic generation of the Ti:sapphire laser output. Narrowband excitation pulses at 500, 550, and 590 nm were obtained using a 400 nm-pumped NOPA, in which the WLC spectrum, generated in a 1 mm-thick sapphire plate and shaped by interference filters to a ±5 nm bandwidth of interest, was employed as the seed. The excitation fluence at different wavelengths was adjusted to achieve a comparable ground state bleaching signal of less than 5 mOD at 500 fs. The measurements presented in the main text are within the low excitation fluence linear regime, unless otherwise mentioned (see the “Fluence dependent transient absorption measurements” section in the Supporting Information).

For the nanosecond TA (ns-TA) measurements, we utilized an amplified femtosecond laser (Light Conversion Pharos, 1024 nm, 300 fs pulse duration, 2 kHz repetition frequency)^44^ to generate broadband probe pulses with spectrum ranging from 510 to 800 nm by WLC in a thin sapphire plate. Electronically synchronized 355 and 532 nm pump pulses, approximately 800 ps in duration, were produced by the third- and second-harmonic of a Q-switched Nd laser (Innolas Picolo), respectively. The excitation fluence was controlled with a neutral density filter to match the signal amplitudes observed in fs-TA at nanosecond time scales.

**Figure 1:**
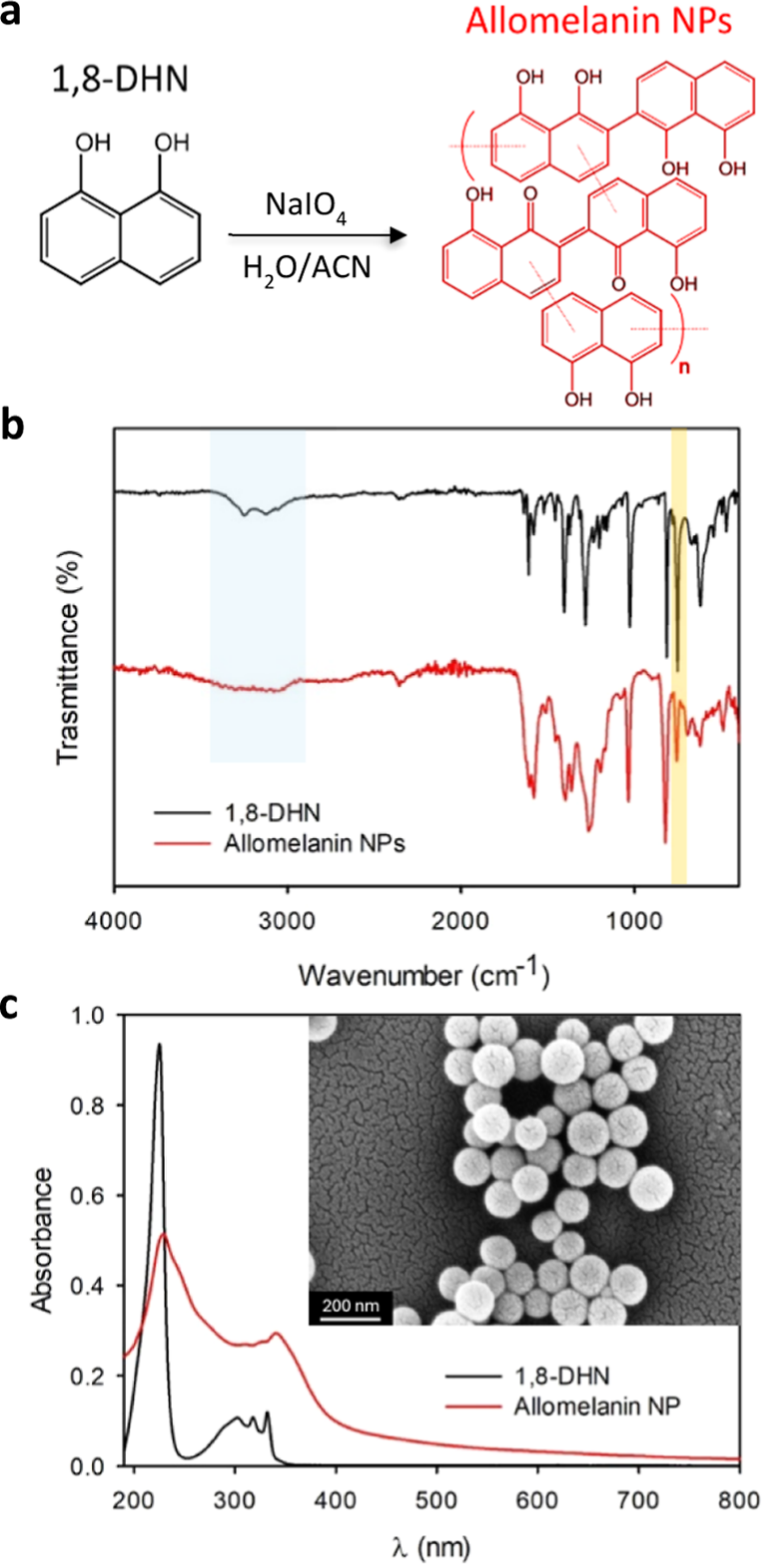
(a) Chemical structure of 1,8-DHN and the oxidation/polymerization procedure used to form allomelanin NPs. Additionally, the currently accepted hypothetical structure of allomelanin NPs is illustrated, consisting predominantly of 1,8-DHN, 2–2′ dimers and their oxidized counterparts, arranged in a π–π stacking pattern. (b) FTIR spectra of 1,8-DHN (black) and allomelanin NPs (red). Key modes are highlighted by shaded rectangles. (c) Absorption spectra of 1,8-DHN (black) and allomelanin NPs in aqueous solvent (red). The inset shows a SEM image of the allomelanin NPs.

A magic angle configuration (54.7°) between the pump and probe polarizations was used in all TA experiments. The samples were prepared in 1 mm-thick quartz cuvettes using water as the solvent. To eliminate concentration-dependent dynamics, the absorbance of allomelanin NPs in water was kept constant across all experiments, with the absorption spectrum shown in Figure 1c. To prevent photodamage within the illuminated area, a continuous flow was maintained during all measurements. Subsequently, global analysis employing multiexponential functions was conducted on the data sets using the Glotaran software.^45^

### Theoretical Calculations

Spectroscopy simulations were performed through a multiconfigurational wave function-based XMS-RASPT2^46^ approach explicitly accounting for the water solvent via a hybrid quantum mechanics/molecular mechanics (QM/MM) setup.^47^ Solvent effects on absorption spectra are quantitatively modeled by averaging the signal over 100 uncorrelated solvent conformations sampled from classical molecular dynamics. All RASPT2 calculations were performed with zero IPEA shift and an imaginary shift of 0.2 employing the cc-pVDZ basis set, and relying on the Cholesky decomposition^48^ to speed up the evaluation of the electron integrals. Extensive details regarding the computational methods are provided in the “Simulations on fundamental allomelanin units” section of the Supporting Information.

## Results and Discussion

### Characterization of Allomelanin NPs

Allomelanin nanoparticles (NPs) in water were synthesized using 1,8-DHN as a precursor and NaIO_4_ as the oxidant, according to the oxidation/polymerization procedure reported in Figure 1a. Figure 1b displays the FTIR spectrum of 1,8-DHN (black) and allomelanin NPs (red). 1,8-DHN exhibits distinct peaks at 3120, 1611, 1402, 1284, and 1038 cm^–1^. These peaks correspond to aromatic C–H stretching, aromatic C=C stretching, C–OH bending, C–OH stretching, and aromatic C–H bending, respectively.^17^ The broad peaks in the 3200–3400 cm^–1^ range (blue shaded rectangle) are attributed to the stretching of –OH and –CH groups on the naphthalene ring, which become broadened in allomelanin due to its inherent geometrical and chemical disorder.^17^ In addition, upon the formation of allomelanin NPs, there is a notable suppression of the aromatic C–H bending peak at 753 cm^–1^ (orange shaded rectangle), indicating intermolecular cross-linking of the naphthalene rings.^17,49^

Detailed chemical characterization of the allomelanin NPs studied in this work is presented in ref (17). Thus, far, the chemical characterization techniques applied to allomelanins collectively reveal that they are primarily composed of oligomers formed by three types of dimers: 2–2′, 4–4′, and 2–4′ (IUPAC numbering).^17,25,50−54^ Among these, the 2–2′ dimer is the most abundant, consistent with prior experimental and theoretical studies.^25,54^ These molecular building blocks, along with their oxidized counterparts,^17,50,54^ self-assemble through hydrogen bonding of –OH groups and π–π stacking of naphthalene rings in aqueous solvents, resulting in the formation of spherical NPs.^17,25^ As a result, the optoelectronic properties of allomelanin can be explained by coupled hydroquinone–quinone pairs.^17^

The chemical characterization of the allomelanin NPs is further complemented by elemental analysis (Table S1). Interestingly, the allomelanin NPs exhibited a relatively higher-than-expected oxygen content compared to the composition initially derived from the 1,8-DHN precursor. This increased oxygen content is primarily attributed to water molecules trapped within the polar functional groups of allomelanin’s building blocks.^55^ In addition, a recent study^55^ proposed that some of this excess oxygen may also be incorporated through ether bridges, potentially contributing to the structural cross-linking of the allomelanin framework.

Figure 1c shows the absorption spectrum and the SEM image of the formed spherical NPs. DLS measurements indicated a hydrodynamic diameter of 170 nm with a polydispersity index of 0.12 (Figure S1). Allomelanin NPs, solubilized in ultrapure Milli-Q water, demonstrated stability over several days. However, when introduced into organic solvents, the NPs disassembled (see discussion in the “Measurements on allomelanin NPs dispersed in thin film, water and organic solvents” section in Supporting Information). Allomelanin in water exhibits a broad and featureless absorption band ranging from the UV to the NIR, contrasting the structured absorption of 1,8-DHN which peaks solely in the UV. Such absorption signature is characteristic of melanins, making them unique among natural biomolecules. Unlike typical organic chromophores possessing structured electronic absorption peaks accompanied by vibronic replicas, melanins resemble the optical properties of amorphous semiconductors.^56,57^

The long absorption tail, covering the visible and extending to the NIR wavelengths, possesses two shoulders at 470 and 580 nm (Figure S3). The 470 nm peak likely arises from excitonic resonances within aggregated NPs, strongly influenced by the NP size and stacking,^17^ becoming more pronounced with increasing NP size or aggregation pattern and diminishing upon deaggregation (see “Measurements on allomelanin NPs dispersed in thin film, water and organic solvents” section in Supporting Information). In contrast, the 580 nm peak, independent of NP size, most likely originates from excitonic transitions in quinoid oligomers,^55^ in agreement with previous laser flash photolysis experiments and theoretical calculations.^54^

Similar to other melanins,^58^ allomelanin exhibits an extremely low fluorescence quantum yield when optically excited in the UV (≈1.5%), which becomes negligible and cannot be precisely determined upon visible (<0.1%) excitation (Figure S1). This observation underscores the dominance of nonradiative excited state relaxation channels, highlighting the efficiency of allomelanin in dissipating absorbed energy nonradiatively.

### Excited State Relaxation Pathways in Allomelanin

**Figure 2:**
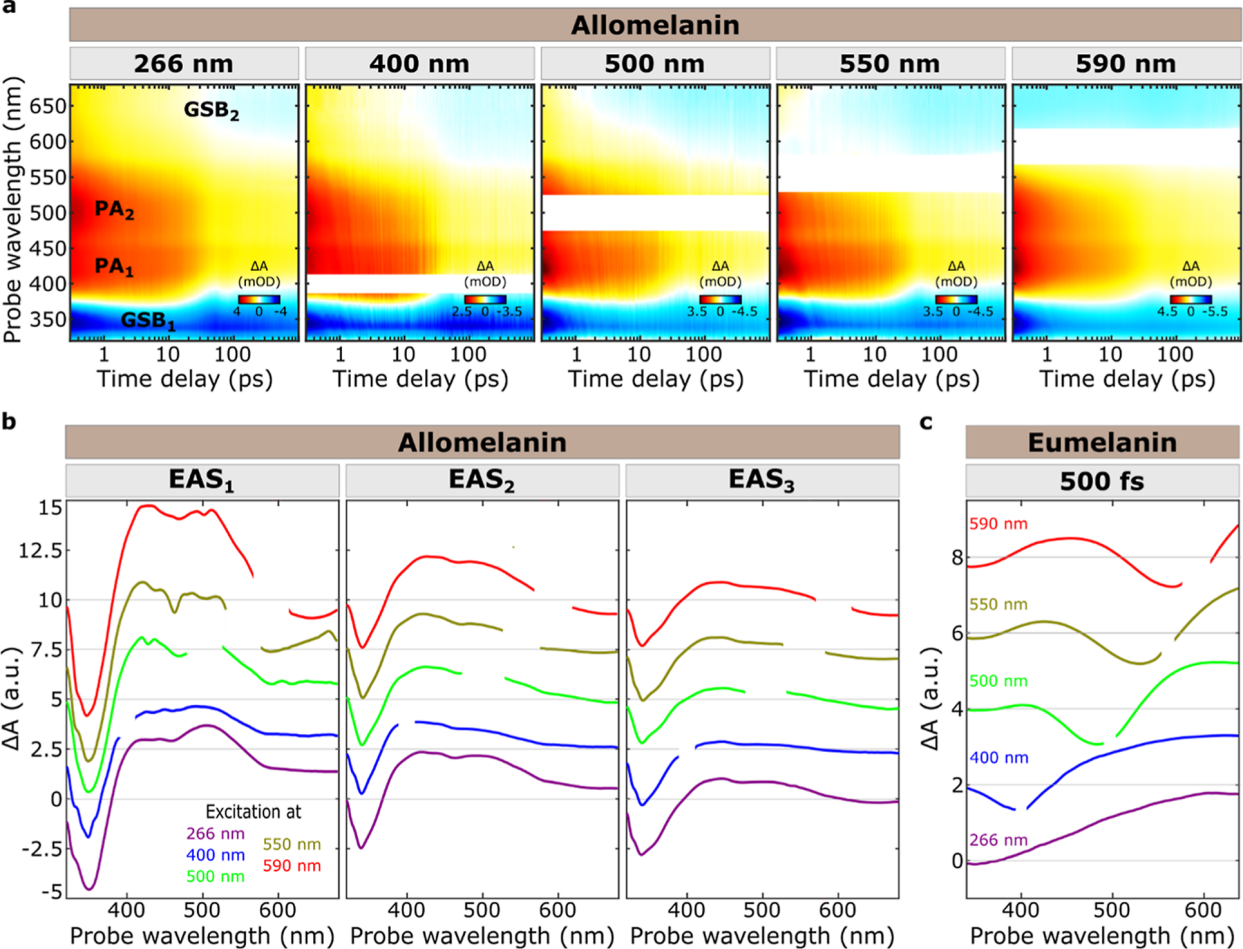
(a) TA maps of allomelanin, as a function of probe wavelength and time, for excitation at 266, 400, 500, 550, and 590 nm (from left to right panel). The EAS spectra of (b) allomelanin obtained after global analysis of the data sets shown in (a), resulting in three EAS components (EAS_1_, EAS_2_, EAS_3_), and the TA spectra of (c) eumelanin obtained at 500 fs. The color coding corresponds to excitation at 266 nm (violet), 400 nm (blue), 500 nm (green), 550 nm (gold), and 590 nm (red). All the data were acquired under magic angle pump and probe relative polarization conditions. The probe region which shows strong pump scattering for each excitation has been omitted.

To map the excited state dynamics in allomelanin, we utilized fs-TA spectroscopy with tunable 100 fs excitation pulses. Excitation at 266 nm (Figure 2a, first panel) induces a ground state bleaching (GSB) signal at 320–370 nm (GSB_1_), accompanied by a structured photoinduced absorption (PA) band extending from 370 to 680 nm. The PA spectrum displays two prominent peaks at 420 nm (PA_1_) and 500 nm (PA_2_), in contrast to the broad and featureless PA band observed around 700 nm in eumelanin-like materials.^7,9,10,59^ In addition, the PA spectrum features two dips at 470 and 580 nm, which align with the allomelanin static absorption peaks in the visible range, suggesting a broad underlying GSB that partially influences the spectral shape of the PA (see Figure S3). This hypothesis is further corroborated by fluence-dependent measurements, where at high excitation fluences annihilation of excited state population unveils the broadband underlying GSB (see discussion in the “Fluence dependent transient absorption measurements” section in Supporting Information). Beyond 100 ps, a new negative Δ*A* signal emerges around 650 nm, which could be associated with GSB and/or to stimulated emission (SE) from the excited state. However, the negligible fluorescence quantum yield at λ > 600 nm (Figure S1) strongly suggests that this signal originates from GSB, and we call it GSB_2_. The appearance of GSB at longer wavelengths relative to the excitation can be attributed to energy transfer to chromophores absorbing at lower energies and/or to the decay of overlapping PA, revealing the underlying GSB response.

**Figure 3:**
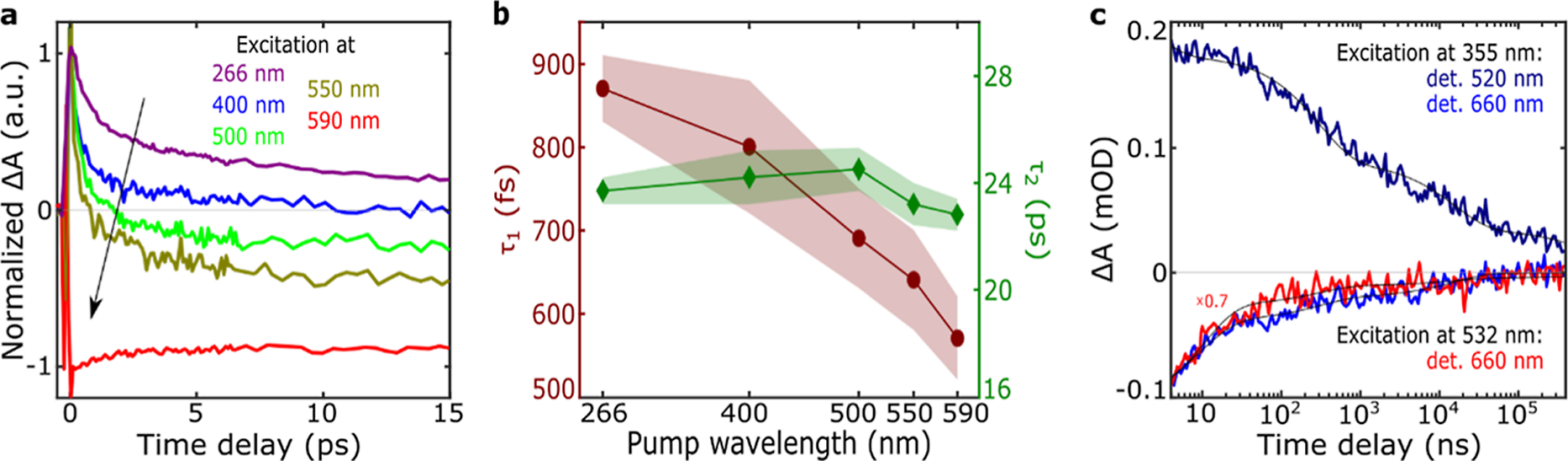
(a) Dynamics at 650 nm, normalized at 0.2 ps, upon excitation at 266 nm (violet), 400 nm (blue), 500 nm (green), 550 nm (gold), and 590 nm (red). (b) Lifetime of the τ_1_ (left axis, maroon) and τ_2_ (right axis, dark green) time components of global analysis, as a function of pump wavelength. (c) Dynamic evolution of the PA peak at 520 nm following 355 nm excitation (dark blue), and the GSB peak at 660 nm following 355 nm (light blue) and 532 nm (red) excitations. The kinetics are recorded for pump–probe delays ranging from 4 ns to 400 μs.

Upon tuning the excitation wavelength to 400, 500, 550, and 590 nm (Figures 2a and S4), a subtly different response is observed, as evidenced by the gradual decrease of the PA_2_ band with respect to PA_1_ immediately after photoexcitation (Figures 2a and S5) and the accelerated formation of GSB_2_ (Figures 2a and 3a). Beyond 100 ps, the system loses its excitation memory, resulting in a universal photoresponse independent of the excitation wavelength. This indicates that the photoexcited population is channeled into a similar distribution of low-energy excited states.

To provide a quantitative analysis of excited state dynamics, we performed a global fitting within the framework of evolution-associated spectra (EAS).^45^ For each excitation wavelength, the data were fitted using a three-excited-species sequential model (Figures S6–S10 and Table S2). The three resulting EAS, shown in Figure 2b, are categorized into two exponential components with sub-100 ps lifetime and a long-lived nondecaying component (≫1 ns). One one hand, the observation of similar PA features in the photoinduced EAS_1_, despite tuning the pump energy over a range of 2.56 eV, underscores the presence of broadband-coupled excited states. On the other hand, the slight deviation between the spectral shapes of EAS_1_ obtained at different excitation wavelengths indicates that photoexcitation leads to a distribution of slightly different excited states, as the system partially retains its excitation memory.

The transition from EAS_1_ to EAS_2_ modifies the TA spectra toward their final shape, which remains consistent upon tuning the excitation wavelength. For 266 and 400 nm excitations, this transition primarily manifests as a decrease in the PA_2_/PA_1_ ratio and a faster decay of the PA at 650 nm (Figures S5 and 3a). For longer wavelengths, the transition predominantly appears as a red-shift of the GSB_2_, as evidenced by the spectral evolution of EAS. This shift is further highlighted by examining the TA signals at early times in the NIR (Figure S11) and red (Figure S13) detection regions upon excitation at 550 and 590 nm, respectively. Overall, the decrease of PA_2_/PA_1_ ratio can be interpreted as a blue-shift of the PA transitions, while the formation/red-shift of GSB_2_ can be seen as a red-shift of the broad GSB. This process, observed throughout the lifetime τ_1_ (Figure 3b, Tables S2 and S3), accelerates as the excitation is tuned to longer wavelengths and corresponds to energy transfer to lower-lying excited states on subpicosecond time scales. The decreasing τ_1_ lifetimes (e.g., from 870 to 570 fs) upon tuning excitation wavelength to the red supports the concept that nonradiative relaxation occurs faster when energy gaps are smaller (Table S3). Although the spectral shapes of all the EAS_2_ are similar, minor deviations between the spectral profiles obtained for different excitation wavelengths may arise from varying relative contributions of the PA to the overlapping GSB.

The subsequent decay of the broadband PA in the visible, as evidenced by the transition from EAS_2_ to EAS_3_ (Figure 2b), better reveals the universal spectral profile of the TA signals. This transition is accompanied by minimal spectral shape changes and no recovery of the GSB, indicating the formation of a new excited state that exhibits weaker PA within our probe spectral window, which we identify as a nonemitting photoproduct. The transition from the initially populated excitonic species (EAS_1_, EAS_2_) to new species with distinct optoelectronic properties (EAS_3_), is supported by fluence-dependent measurements (see “Fluence dependent transient absorption measurements” in Supporting Information). Interestingly, the second lifetime τ_2_ (23.7 ± 0.9 ps; Figure 3b and Table S2), associated with the population of the long-lived photoproduct, remains nearly unaffected by the excitation wavelength. This further supports the hypothesis of downhill energy transfer toward a distribution of low-energy states.

For completeness, we report the effects of NP morphology (size and packing) and the solvent environment on the ultrafast photophysics of allomelanin. TA measurements of allomelanin NPs dispersed in thin films, water, and organic solvents, with detailed results provided in the “Measurements on allomelanin NPs dispersed in thin film, water and organic solvents” section in Supporting Information, highlight the presence of similar spectral signatures in different environments. However, in thin films, densely packed NPs exhibit faster kinetics compared to those in water, driven by enhanced intermolecular interactions. In contrast, disaggregated NPs in organic solvents show the slowest kinetics. Overall, NP size and packing predominantly dictate kinetic behavior, while the solvent environment primarily influences aggregation states.

Next, we examine the kinetics of the long-lived photoproduct following 355 and 532 nm excitation using ns-TA spectroscopy (Figure 3c). The TA spectra exhibit features similar to those observed at subnanosecond pump–probe delays, indicating the persistence of long-lived species from 24 ps to >450 μs (Figures S14 and S15; Table S4). Both data sets were fitted with biexponential decays (310 ns and 15.8 μs) and a long-lived nondecaying component extending beyond our temporal observation window (>450 μs). The slightly varying amplitudes reveal that UV excitation produces residual long-lived signals with twice the amplitude observed under visible excitation. Overall, the similarity of kinetics across excitation wavelengths further support the ultrafast energy transfer to a similar distribution of states, regardless of the initial excitation wavelength.

Additionally, to demonstrate that these long-lived signals lead to the accumulation of stabilized photoproducts under continuous light exposure, we performed a time-resolved absorption experiment on the seconds-to-minutes time scale using LED excitation at 400 nm (for details see “Reversible changes in the absorption of allomelanin NPs upon irradiation” section in Supporting Information). By tracking the absorbance of allomelanin NPs at 850 nm, we observed an exponential increase over approximately 40 s during light irradiation, attributed to the formation of long-lived photoproducts. After 600 s, the light was switched off, and the process was fully reversible, with the absorbance decaying exponentially over approximately 60 s.

In addition to the excited-state dynamics discussed above, another important aspect of Figure 2 is the GSB response of allomelanin compared to reference experiments on eumelanin, which shows a GSB following the excitation wavelength (spectral hole burning). Details of the experimental conditions for eumelanin NPs (Figure 2c; “Measurements on the eumelanin analogue polydopamine” section) and a comprehensive comparison of the key features observed in allo- and eu-melanins (“Comparison of allomelanin’s and eumelanin’s key features” section) can be found in the Supporting Information.

The origin of the broadband and featureless absorption spectrum of melanin-like materials remains debated, with proposed models ranging from the superposition of weakly interacting distinct absorbers^31,56,60,61^ to the presence of strongly coupled chromophores.^8,30,33,62^ Pioneering experimental studies on eumelanin-like materials have revealed the presence of strongly coupled chromophores, although these interactions are seemingly confined within the excited subset of chromophores.^7,8,30^ Specifically, the selective excitation of energetically proximal coupled chromophores in eumelanin leads to a GSB signal that manifests itself as a spectral hole burnt over the broad TA spectrum (Figure 2c). A similar response has been observed in TA experiments of disordered carbonaceous nanomaterials and similarly attributed to their pronounced spectral heterogeneity.^34−37^

In contrast, allomelanin exhibits a notable absence of wavelength-dependent spectral hole-burning. Here, strongly coupled chromophores distribute the couplings across a broad energy spectrum, as evidenced by the GSB signal, which closely mirrors the features of allomelanin’s absorption spectrum (see “Fluence dependent transient absorption measurements” in Supporting Information). Furthermore, the similarity of the GSB and PA responses, regardless of the excitation wavelength, underscores the presence of collective excitations within strongly coupled chromophores, facilitating the efficient funneling of the absorbed energy to lower-energy species. This represents the first direct observation of a melanin-like material exhibiting broadband couplings across the UV to visible spectrum.

### EPR under Irradiation

**Figure 4:**
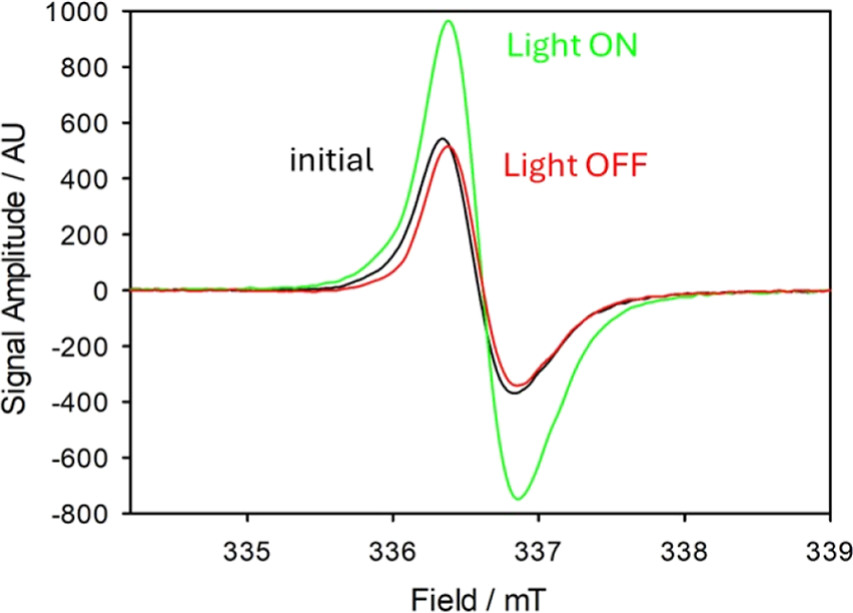
EPR spectra of allomelanin NPs in water at 30 °C, in the dark (black) upon irradiation (green) and upon switching the irradiation off again (red), showing reversible spectral changes upon UV irradiation of the sample.

X-band EPR spectra of allomelanin NPs were measured before, during, and after irradiation in water suspension at 303 K, as shown in Figure 4. These measurements demonstrate that the long-lived species observed in the transient absorption experiments (from nanoseconds to microseconds to seconds) are directly associated with an increment of the radical content of melanins.^59^

Before irradiation, the EPR spectrum of allomelanin NPs showed the typical broad band with unresolved hyperfine structure, resulting in a line width of about 5 G (0.5 mT) (Figure 4; black line). The signal was centered at *g* = 2.0033 to 2.0034, in good agreement with previous studies on DHN-derived allomelanins,^50,51,63^ and other melanins.^6,64−67^ As previously reported,^64,65,68,69^ this EPR signal likely arises from the superposition of different radical species present in dark conditions, e.g., a prevailingly C-centered radical species with *g* = 2.0032 and a semiquinone-type O-centered radical with *g* = 2.0045. Environmental factors such as the surrounding medium,^64^ particularly ionic liquids,^63,65,66^ and light irradiation^70^ can alter the spin distribution within these species, shifting their equilibrium, thereby affecting the g-value, the spectral line width and signal intensity.

Under light exposure of melanins in water, proton-coupled electron transfer (PCET) from hydroquinone-type to their fully oxidized counterparts, of quinone-type structures, shifts the comproportionation equilibrium (see “Light-induced comproportionation equilibrium schemes” section in Supporting Information), forming intermediate protonated semiquinone-type (SQH) radicals.^70,71^ The pH of the aqueous environment determines the formation rate of the anionic semiquinone-type radicals (SQR), which dominate the light-induced EPR spectra at physiological pH (∼7).^70,71^

**Figure 5:**
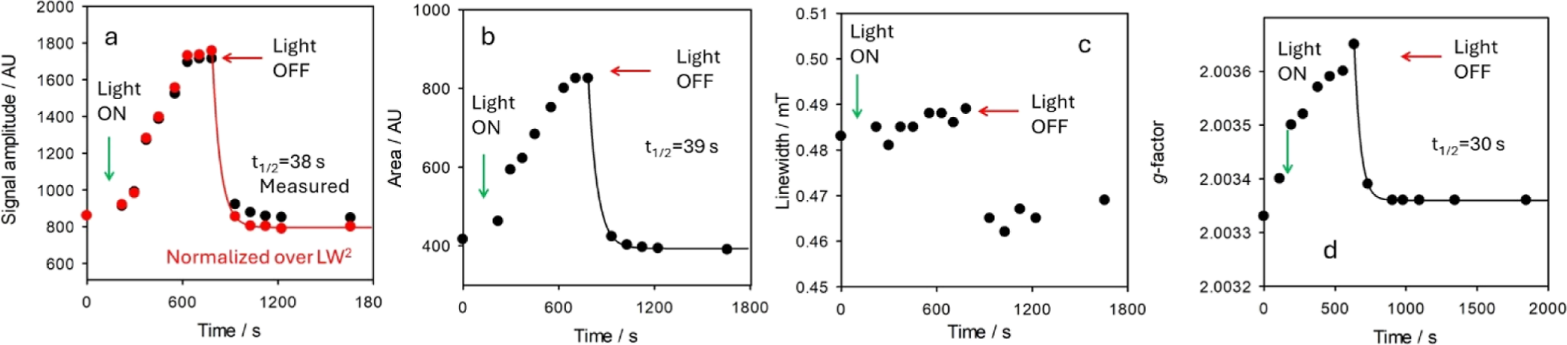
EPR spectral parameters of allomelanin NP in water at 30 °C, upon irradiation showing: (a) the EPR signal amplitude, (b) the radical concentration, (c) the spectral line width, and (d) the *g*-factor. The time points of irradiation are indicated and the decay is fitted using an exponential equation.

Here, we show that the electron paramagnetic properties of allomelanin are reversibly modified by UV irradiation. EPR spectra recorded at 60 s time intervals on allomelanin samples, contained in a quartz tube and subjected to continuous irradiation with a W–Hg lamp (200–400 nm) directly in the cavity of the EPR spectrometer, revealed significant changes upon irradiation (Figures 4 and 5). Both signal intensity and the radical concentration in the sample, as assessed by the double integral of the EPR signal, progressively increased up to a plateau value (Figure 4; green line). These changes were accompanied by a broadening of the spectral line width which was also substantially shifted to lower field, implying an increase in the *g*-factor. Interestingly, upon shutting off the irradiation, the spectral changes rapidly reversed, progressively regaining features close to the original state before irradiation (Figure 4; red line). The time course of variation of the main spectral parameters upon irradiation is shown in Figure 5, where the reversible nature of changes upon interrupting irradiation is clearly visible. The EPR signal intensity decays as a first order process (exponential decay), yielding a half-life of about 40 s. A similar time-course was observed for *g*-value and line width which were restored to nearly the initial values.

In line with previous observations with different melanins including allomelanin in different ionic liquids,^63,65,66^ these EPR spectral changes can be attributed to a reorganization of the electronic properties of allomelanin, reflecting shifts in the equilibrium among different radical species. Irradiation likely promotes the light-driven formation of SQR-like oxygen-centered radicals. We note that oxygen-centered semiquinone-like radicals are more stable and generally less reactive than carbon-centered radicals due to electron delocalization, which supports their role in protecting cells against oxidative stress.^72^ When the light source is switched off, these products deplete and revert to the hydroquinone–quinone mixture, resulting in a corresponding decrease in the observed free radical population.^13,70^

### Characterization of the Long-Lived Photoproduct

As previously mentioned, a simple redox equilibrium model involving quinone- and hydroquinone-like structures has been proposed^70,71^ to explain the extrinsic radicals observed in the light-dependent EPR signals of melanins, corresponding to species detectable on microsecond to second time scales. Complementary to EPR, TA spectroscopy captures photophysical processes occurring on femtosecond to microsecond time scales. The TA spectral features of melanins have similarly been interpreted through light-driven electron transfer within hydroquinone–quinone pairs in melanin aggregates, leading to the generation of charge-transfer (CT) states on the ultrafast time scale,^8,9,59,73^ which may be further stabilized through proton transfer reactions.^10,59,74^

However, EPR and TA experiments in melanins are typically treated separately due to their inherently distinct time scales and sensitivity, leaving a unified model yet to be established. Recently, Grieco et al.,^59^ suggested the picosecond formation of semiquinone-like photoproducts via photoinduced comproportionation. Nevertheless, the authors highlighted the lack of reported absorption spectra for melanin radicals in the literature, making definitive spectral assignments from experimental TA data sets challenging.

**Figure 6:**
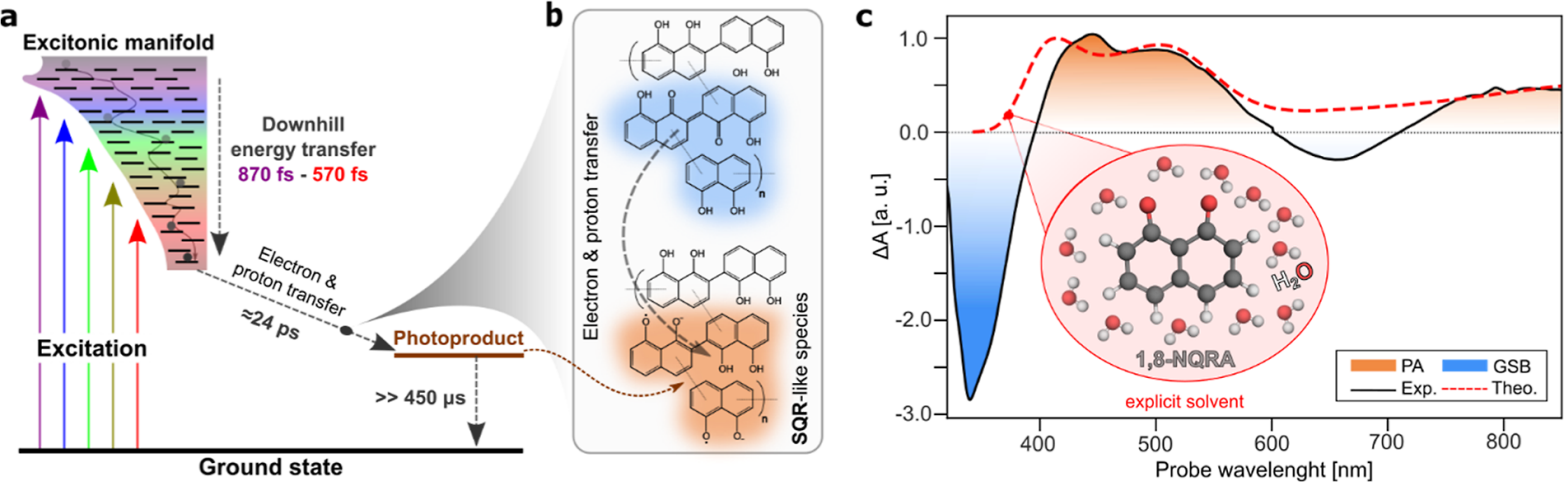
(a) Schematic interpretation of the relaxation dynamics after photoexcitation of allomelanin. An initial downhill energy transfer process occurs within 570–870 fs, depending on the excitation wavelength. This is followed by electron and proton transfer reactions (illustrated in (b)) between hydroquinone- and quinone-like pairs on a time scale of 24 ps, resulting in the formation of long-lived (>450 μs) SQR-like species. In Figure 6a, the black lines represent electronic transitions within the excitonic manifold, composed of allomelanin’s subspecies, which are strongly coupled, as indicated by the absence of spectral hole-burning effects and the broadband GSB transitions. (c) Comparison of experimental and theoretical spectra supporting the hypothetical photochemical pathways. Hybrid QM/MM XMS-RASPT2 simulations (red dashed line) attribute the experimentally observed PA features of the long-lived TA signal (solid line with shaded area underneath; represented by the EAS_3_ component of the global analysis upon 266 nm excitation) to the absorption of the water-solvated 1,8-NQRA photoproduct (shown in inset)—the minimal unit of SQR-like species likely formed in 1,8-DHN-derived allomelanin. A red shift of 0.2 eV was applied to the theoretical results to better align them with the experimental data.

In allomelanin, building on the hydroquinone–quinone redox model, a plausible mechanism involves the funnelling of initially absorbed energy to a comparable distribution of low-energy species within 570–870 fs (Figure 6a), followed by the formation of CT states between hydroquinone–quinone pairs (Figure 6a,b; see “Proposed photochemical pathways in allomelanin” in Supporting Information). Parallel competing pathways include the formation of bound SQR-derived CT states and the stabilization of SQR-like separated charges through proton transfer processes, facilitated by hydrogen-bonding networks and the close spatial arrangement of redox-active groups found in allomelanin (Figure 6a,b; see “Proposed photochemical pathways in allomelanin” in Supporting Information).

To further test our hypothesis, we have modeled the spectroscopic features associated with SQR-like species using multiconfigurational wave function-based XMS-RASPT2^46^ theory in a QM/MM setup.^47^ As a first approximation, we model the complex molecular structure of SQR-like species with their fundamental unit in 1,8-DHN-derived allomelanin, the 1,8-naphthoquinone radical anion (1,8-NQRA; inset of Figure 6c) solvated in water.

The absorption spectrum for water-solvated 1,8-NQRA was calculated by averaging the signal over 100 uncorrelated solvent snapshots extracted from classical molecular dynamics, enabling a quantitatively accurate description of solvent effects. Three main peaks can be identified in the simulated spectrum (red dashed line in Figure 6c), namely: (i) a very broad absorption centered in the NIR region (around 800 nm) extending toward the visible region of the spectrum; (ii) two sharper absorption peaks at 520 and 450 nm, respectively. A natural transition orbital (NTO) analysis^75^ displaying the character of the electronic transitions that give rise to the 1,8-NQRA absorption spectrum is shown in Figure S33 of the Supporting Information. Further details about the simulation protocol are given in “Simulations on fundamental allomelanin units” section of Supporting Information.

In TA spectroscopy, the absorption spectrum of photoproducts appears as PA transitions. Comparing the PA transitions of the long-lived photoproduct (Figure 6c; solid line; orange shaded area) with those of the 1,8-NQRA unit provides key mechanistic and structural insights. Despite the structural complexity of allomelanin, the agreement between the experimental TA spectrum, represented by the EAS_3_ component of the global analysis upon 266 nm excitation, and the calculated spectrum for 1,8-NQRA suggests that the formed anionic radicals are highly localized and can be accurately described by SQR-like monomeric signatures.

Quantum chemistry calculations further reveal that this localization is resilient to the inherent disorder in allomelanin aggregates, whether chemical or conformational in origin, which can alter site energies and deviate significantly the PA transitions from a monomeric response. Such disorder may manifest in allomelanin as intra- or interchain heterogeneity. While interchain effects are beyond the scope of this study, the minimal impact of intrachain heterogeneity can be explained by steric repulsion between hydroxyl groups on adjacent naphthyl units. This interaction induces a pronounced tilt angle (close to 70°; see Figure S34 in the Supporting Information) between the two cyclic subunits of the most abundant 2–2′ dimer-derived SQR species, effectively decoupling their conjugation.

Having established the chemical identity of the long-lived photoproduct as predominantly localized SQR-like species, we estimate the relative quantum yield of radical formation under 266 and 550 nm excitation based on the PA amplitude of the photoproduct. Remarkably, UV excitation produces a 4-fold higher radical yield compared to visible excitation (see “Estimation of radical quantum yields through TA measurements” in Supporting Information).

Furthermore, given the SQR-like chemical character of the radicals, we comment on their long-lived behavior presented in Figures 3c and 6a. Catechol-quinone dimeric model systems of eumelanin, where semiquinone-like photoproducts are formed via PCET, typically exhibit biexponential decay kinetics attributed to direct and diffusion-controlled recombination pathways.^76^ In melanins, due to their inherent disorder, the complex dynamics is usually approximated by up to four exponential terms.^9,77,78^

In this context, we assign the observed biexponential decay in allomelanin, with an average lifetime of 6.6 μs, to recombination pathways influenced by variations in SQR-derived CT distances and their associated electronic couplings, shaped by molecular conformations and the local environment. The long-lived (≫450 μs), nondecaying plateau component is attributed to stabilized, charge-separated SQR-like species.

On the longer seconds-to-minutes time scale, the light-dependent absorption underscores the dynamic adaptability of allomelanin NPs under illumination, driven by the reversible formation and recombination of SQR-like radical species, which ultimately contribute to the EPR-detected radicals. Furthermore, as shown in Figure S24, these species recombine reversibly in the presence of oxygen, ruling out the formation of intermediate triplet species (for detailed investigation of possible triplet states in allomelanin see the “Investigation of intermediate triplet states in allomelanin” section in the Supporting Information).

Last but not least, the unified mechanistic model presented here explains the formation of SQR-like species detectable by the complementary techniques of TA and EPR. While both techniques attribute their features to species of similar SQR-like nature, those observed in TA may undergo interconversion or structural reorganization into a more thermodynamically stable form before being detected by EPR, a limitation imposed by the resolution of our experimental techniques. This underscores the complexity of the system and the dynamic evolution of radicals in allomelanin, highlighting the need for further investigation to fully resolve the intermediate processes.

### Light-Enhanced Antioxidant Activity of Allomelanin

Light-driven formation of semiquinone-like species has similarly been reported in previous studies on eumelanins and is suggested to be central to their physiochemistry and biochemistry.^13,70,79,80^ The potential role of these photoactive, rapidly reversible species in redox reactions remains unclear. They have been proposed as a means to quench harmful radicals, although concerns have been raised about their potential phototoxicity in living organisms.^70,81^

Here, we demonstrate that in allomelanin NPs, the photoinduced increase in radical content and the shift to SQRs can significantly enhance their antioxidant activity upon light exposure. To address this property, we performed radical scavenging measurements for allomelanin both in the dark and under light exposure. The first set of experiments was carried out upon irradiation with artificial light, using a violet LED (400 nm wavelength), while the second series of experiments was performed under ambient solar light irradiation.

Quenching of the model DPPH was used to indicate the potential antioxidant (AOX) activity. DPPH degradation was monitored by measuring its absorbance at 517 nm within 30 min after the mixing with allomelanin NPs (see Experimental Section). Since DPPH degradation is partially activated by light, irradiation was controlled to ensure less than 10% photodegradation of DPPH. The irradiated radical was then used as a reference for determining AOX activity.

**Figure 7:**
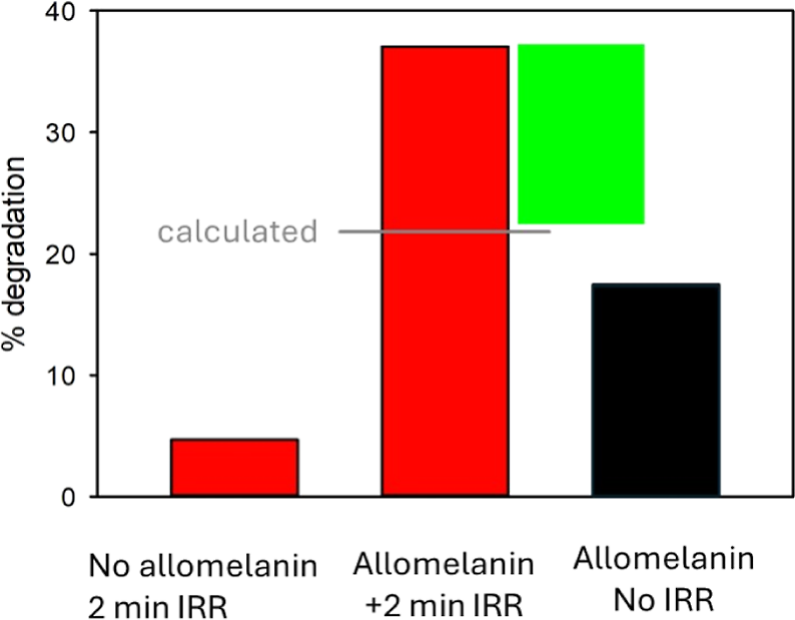
Percentage of degradation of the model radical DPPH upon irradiation with an LED emitting at 400 nm (left) or in the dark in the presence of allomelanin NPs at concentration 7.4 μg mL^–1^ (right). The combined presence of allomelanin NPs 7.4 μg mL^–1^ and irradiation with light produces a DPPH degradation (central red bar) much higher than expected by adding the left and right column (gray line). The excess of degradation (green bar) is due to the synergistic effect of light and allomelanin.

Results for artificial irradiation (400 nm) are shown in Figure 7. In line with previous studies,^25,63^ allomelanin shows AOX activity even in the absence of irradiation. Quenching of DPPH is about 17% for a concentration of 7.4 μg mL^–1^ of allomelanin NPs. Interestingly, irradiation of allomelanin for 2 min at 400 nm increases the DPPH degradation to 37%. Since DPPH is itself slightly photosensitive, we also irradiated it under the same experimental conditions, observing a degradation of about 5%. Hence, the percentage of DPPH loss observed by irradiating allomelanin NPs is about double what is expected by simply adding the effects of light and allomelanin NPs on DPPH (expected 22%). This result aligns with the photoinduced increase in the EPR signal amplitude upon irradiation, and thus the increase in the concentration of radical species, as shown in Figure 5. In conclusion, allomelanin NPs are not only able to efficiently absorb UV-to-NIR light, as shown in Figure 1, but they can utilize the absorbed energy to enhance their AOX activity.

**Figure 8:**
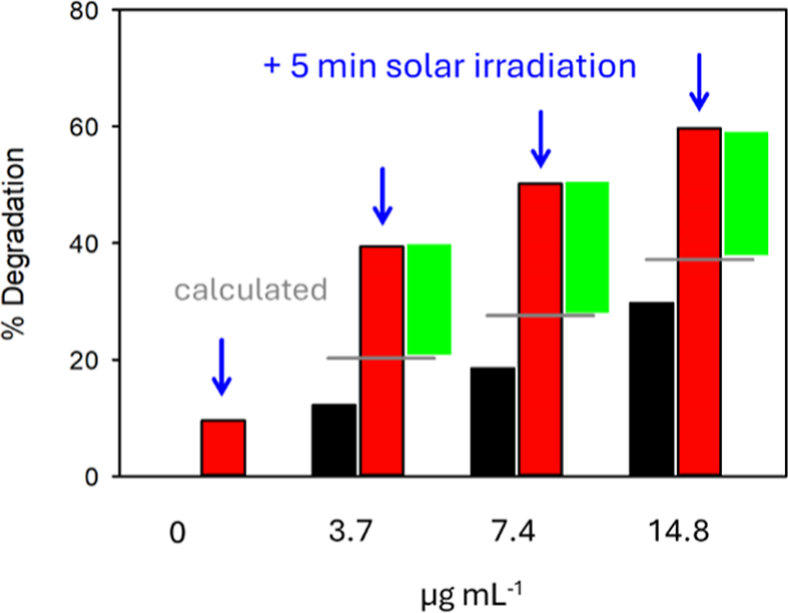
Percentage of degradation of the model radical DPPH by allomelanin NPs without irradiation (black bars) and after 5 min exposure to solar light (red bars). Gray lines show the percentage of degradation calculated by adding the degradation expected because of the light exposure of DPPH and the presence of allomelanin. Green bars show the excess of degradation experimentally observed and resulting from the synergistic effect of light and allomelanin.

To demonstrate that this “synergistic” photoprotective activity is present also in “real” conditions we repeated the radical scavenging activity experiment under ambient sunlight exposure. The results, shown in Figure 8, further demonstrate that exposure of allomelanin to solar light causes an increase of its radical scavenging activity. Focusing on the sample with the lowest allomelanin concentration (3.7 μg mL^–1^), as shown in Figure 8, it is observed that 5 min of exposure to solar light produce a degradation of DPPH of about 40%. This is approximately double the expected degradation, considering separately the effects of irradiation (10% degradation) and allomelanin (12%) on DPPH.

## Conclusions

Allomelanin NPs, derived from the oxidation/polymerization of 1,8-DHN, exhibit excitonic couplings between electronic states spanning UV to NIR energies, significantly deviating from the well-documented transient optical spectral heterogeneity observed in eumelanin analogues and the—spectroscopically related-disordered carbonaceous nanomaterials. Under tunable excitation from UV to visible wavelengths, allomelanin efficiently channels absorbed energy into similarly distributed low-energy excited states on the subpicosecond time scale, with the downhill energy transfer accelerating as the excitation wavelength shifts to the red. Within 24 ps, regardless of the excitation wavelength, energy is funneled into photoproducts with prolonged lifetimes (≫450 μs). Supported by quantum chemistry calculations, we attribute these long-lived states to predominantly localized 1,8-NQRA, SQR-like photoproducts, formed through electron and proton transfer reactions between hydroquinone- and quinone-like pairs. Alongside, the calculations provide structural insights into the complex allomelanin aggregates, resulting in the mainly localized excited state distribution.

Furthermore, EPR and radical scavenging assays experiments reveal that the light-enhanced concentration of SQR-like species significantly boosts allomelanin’s antioxidant activity under solar irradiation. Methodologically, this work aims to bridge the gap between TA and EPR studies, proposing a unified model to explain the photophysical and photochemical behavior of allomelanin. Our results suggest that the two key features of allomelanin—its ability to absorb light across a wide range of wavelengths and to scavenge radicals—can be synergistically coupled to enable new functions in applications such as nanomedicine, energy storage and conversion, and environmental remediation.

## Supporting Information Available

Additional details on measurements of the eumelanin analogue polydopamine, characterization of allomelanin NPs, and TA measurements of allomelanin NPs, including global analysis and fluence-dependent TA measurements. Further investigations cover TA measurements on allomelanin NPs dispersed in thin films, water, and organic solvents, as well as reversible changes in absorption upon irradiation. Other key topics include the investigation of intermediate triplet states in allomelanin, a comparative table of allomelanin’s and eumelanin’s key features, and light-induced comproportionation equilibrium schemes. Additionally, proposed photochemical pathways in allomelanin, estimation of radical quantum yields through TA measurements, and simulations on fundamental allomelanin units are provided. (PDF)

## Abbreviations

1,8-DHN: 1,8-dihydroxynaphthalene
1,8-NQRA: 1,8-naphthoquinone radical anion
AOX: antioxidant
CT: charge transfer
DLS: dynamic light scattering
DPPH: 2,2-diphenyl-1-picrylhydrazyl
EAS: evolution associated spectra
EPR: electron paramagnetic resonance
FTIR: Fourier transform infrared
GSB: ground state bleaching
NP: nanoparticle
PA: photoinduced absorption
PDA: polydopamine
PCET: proton-coupled electron transfer
QM/MM: quantum mechanics/molecular mechanics
SEM: scanning electron microscope
SE: stimulated emission
SQH: protonated semiquinone radical
SQR: semiquinone radical anion
TA: transient absorption
