# Supplementary material for: Photochemical Pathways and Light-Enhanced Radical Scavenging Activity of 1,8-Dihydroxynaphthalene Allomelanin
Source: J Am Chem Soc. 2025 Mar 7;147(11):10031–43. doi: 10.1021/jacs.5c01855 (PMC11926873; doi:10.1021/jacs.5c01855)
Supplement: Supplementary file 1 — ja5c01855_si_001.pdf [file ja5c01855_si_001.pdf]

SUPPORTING INFORMATION

Photochemical Pathways and Light-Enhanced Radical Scavenging Activity of 1,8-dihydroxynaphthalene Allomelanin

Vasilis Petropoulos[a], Dario Mordini[b], Francesco Montorsi[c], Mert Akturk[a], Arianna Menichetti[b], Andrea Olivati[d], Annamaria Petrozza[d], Vittorio Morandi[e], Margherita Maiuri[a], Nathan C. Gianneschi[f], Marco Garavelli[c], Luca Valgimigli[b], Giulio Cerullo[a*], and Marco Montalti[b*]

[a]Dipartimento di Fisica, Politecnico di Milano, Piazza Leonardo da Vinci 32, Milano, Italy.

[b]Department of Chemistry "Giacomo Ciamician", University of Bologna, Via Selmi 2, Bologna 40126, Italy.

[c]Dipartimento di Chimica industriale "Toso Montanari", Università di Bologna, via Piero Gobetti 85,40129 - Bologna, Italy.

[d]Center for Nano Science and Technology @PoliMi, Istituto Italiano di Tecnologia, Via Rubattino 81, 20134 Milan, Italy

[e]Istituto per la Microelettronica e i Microsistemi (IMM), Consiglio Nazionale delle Ricerche (CNR), via Gobetti 101, Bologna 40129, Italy

[f]Departments of Chemistry, Materials Science & Engineering, Biomedical Engineering and Pharmacology, Northwestern University, Evanston, Illinois60208, United States; Department of Chemistry &Biochemistry, University of California San Diego, La Jolla, California 92093, United States

Table of Contents

1. Measurements on the eumelanin analogue polydopamine......................S3

2. Characterization of allomelanin NPs S4

2.1 Dynamic light scattering and emission spectroscopy of allomelanin S4

2.2 Light scattering efficiency of the allomelanin NPs S5

2.3 Stability of allomelanin NPs in water..S6

2.4 Elemental analysis of the allomelanin NPs and 1,8-DHN samples......S6

3. Transient absorption spectroscopy of allomelanin S6

3.1 Global analysis on fs-TA datasets..S10

3.2 Red-shift of the red-edge ground state bleaching signal...................S15

3.3 Global analysis on ns-TA datasets...S18

4. Fluence dependent transient absorption measurements........................S20

5. Measurements on allomelanin NPs dispersed in thin film, water and organic solvents S23

6. Reversible changes in the absorption of allomelanin NPs upon irradiation S29

7. Investigation of intermediate triplet states in allomelanin S30

8. Comparison of allomelanin's and eumelanin's key features S32

9. Light-induced comproportionation equilibrium schemes.......................S33

10. Proposed photochemical pathways in allomelanin S34

11. Estimation of radical quantum yields through TA measurements S36

12. Simulations on fundamental allomelanin units S38

13. References S43

1. Measurements on the eumelanin analogue polydopamine

Polydopamine nanoparticles synthesis

Polydopamine (PDA) nanoparticles (NPs) were synthesized through the spontaneous oxidation of dopamine (DA) hydrochloride in aerobic and alkaline conditions, following a previously established method.[1,2] Briefly, 40 mL of ethanol and 90 mL of deionized water were combined in a 250 mL round-bottom flask. Then, 2 mL of 28-30% NH_4OH were added to the flask, and the mixture was stirred vigorously for 10 minutes. Next, a DA solution was prepared by dissolving 400 mg of DA hydrochloride in 10 mL of deionized water. This DA solution was rapidly injected into the flask under vigorous stirring, resulting in a final DA concentration of 14.9 mM. An immediate color change from light yellow to dark brown was observed, evolving over time. The reaction mixture was stirred vigorously for 24 hours. After this period, the batch was centrifuged to isolate the formed NPs. Specifically, the NPs were collected by centrifugation at 14,000 rpm for 20 minutes, then washed three times with deionized water at 10,000 rpm, and finally redispersed in the same solvent.

Transient absorption measurements of eumelanin

As highlighted in previous studies, PDA serves as a biomimetic analogue to eumelanin.[2–4] To facilitate a direct comparison with the photophysics of allomelanin, we conducted transient absorption (TA) measurements on PDA nanoparticles dispersed in water solvent using 100-fs excitation pulses centered at 266 nm, 400 nm, 500 nm, 550 nm, and 590 nm. Throughout these measurements, the concentration of the solution was kept constant, and the PDA NPs were circulated using a pump to prevent photodamage. The pump fluence was adjusted to ensure a similar ΔA signal at 500 fs across all excitation wavelengths, maintained below 5 mOD. Consistent with existing literature on eumelanin derivatives,[2,5–7] the TA data for PDA NPs revealed wavelength-dependent hole burning, which is assigned to their chemical heterogeneity.

2. Characterization of allomelanin NPs

2.1 Dynamic light scattering and emission spectroscopy of allomelanin

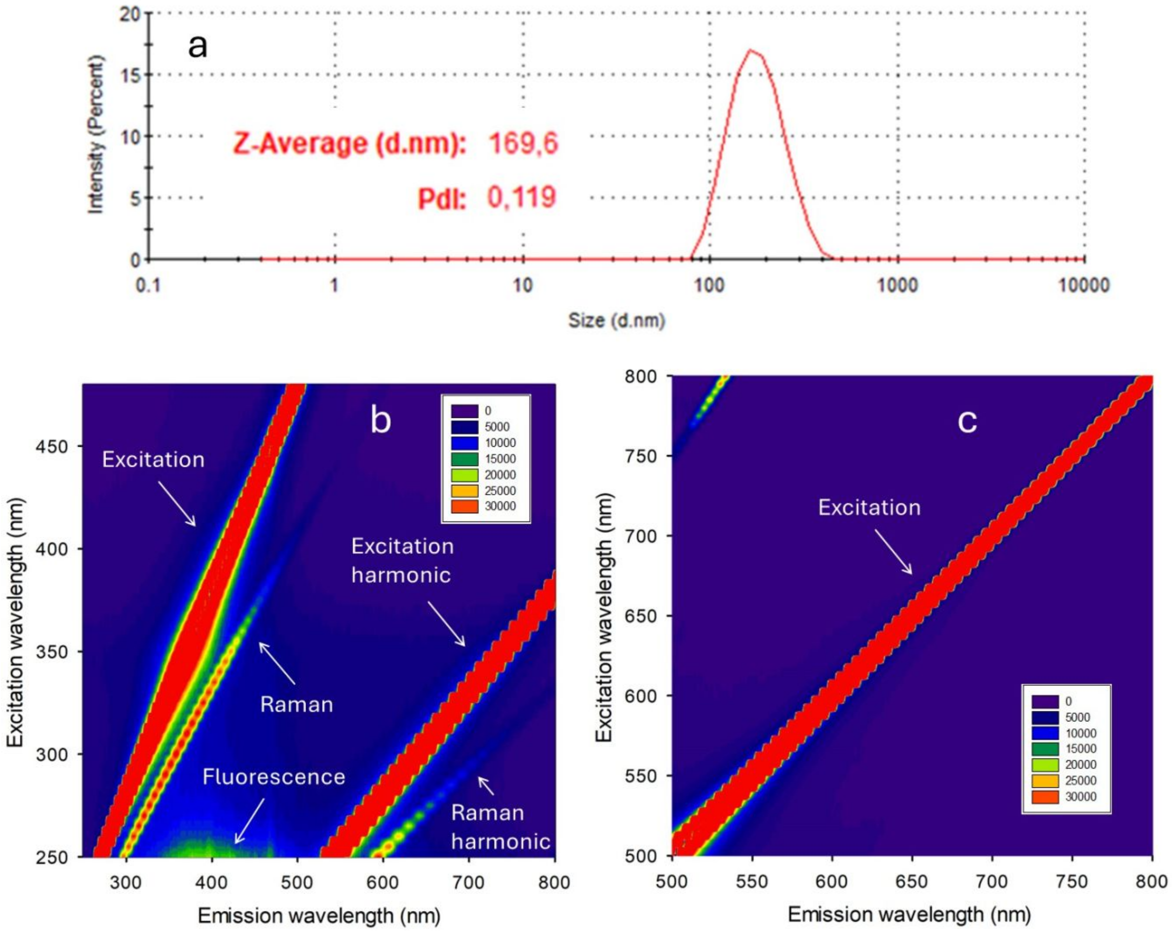

Figure S1: **(a)** Dynamic light scattering (DLS) size distribution of allomelanin, highlighting the average hydrodynamic diameter (Z-average) and the polydispersity index (PDI) values of the formed NPs. **(b)** 2D excitation-emission maps of allomelanin NPs, obtained upon excitation in the wavelength range of 250-500 nm and detecting the emission at 250-800 nm, in quartz cells with a path length of 1.0 cm. The spectra show low-intensity emission between 350-450 nm, while in the other spectral ranges, the emission is negligible. The additional peaks can be attributed to excitation wavelength-dependent signals such as the light-source of excitation, the Raman peak of water, the excitation harmonic, and the Raman harmonic of water. **(c)** 2D excitation-emission map upon excitation in the 500-800 nm range. Only the excitation peak can be detected. As mentioned in the main text, the emission quantum yield of allomelanin NPs for UV excitation (< 320 nm) is 1.5%. However, this value may include contributions from unreacted monomeric 1,8-dihydroxynaphthalene residuals, which could increase the overall yield. For visible excitation (400-500 nm), where monomeric contributions are absent, the emission quantum yield is so low that it cannot be precisely determined (< 0.1%).

2.2 Light scattering efficiency of the allomelanin NPs

The scattering efficiency (quantum yield) of a suspension of allomelanin NPs was determined using silica NPs (Ludox AS-40, Sigma-Aldrich) as a reference. The intensity was measured using a Fluoromax-4 fluorimeter by performing a simultaneous excitation-emission scan. The quantum yield was then calculated according to the following equation:

$$\Phi = \frac{I_{allom}(\lambda)}{A_{allom}(\lambda)} \times \frac{A_{SiO2}(\lambda)}{I_{SiO2}(\lambda)}$$

The wavelength-dependent scattering quantum yield is presented in Figure S2.

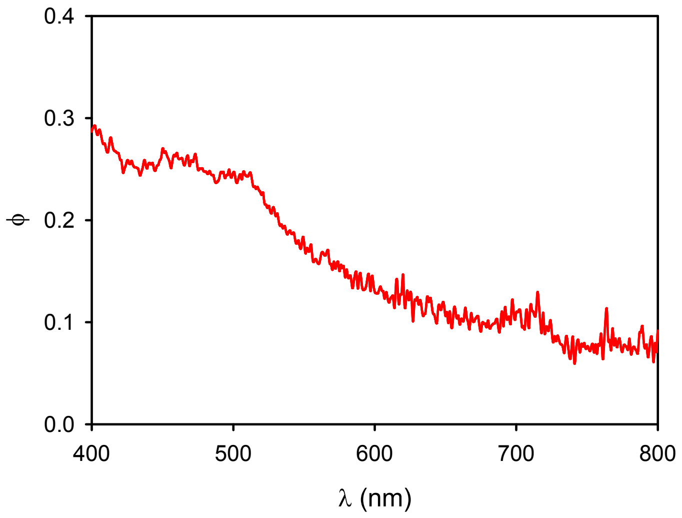

Figure S2: Light scattering quantum yield of a water suspension of allomelanin NPs.

2.3 Stability of allomelanin NPs in water

The stability of allomelanin NPs in water was assessed DLS. A freshly prepared suspension was analyzed one hour after preparation, and the same suspension was reanalyzed after three days. No significant change in the hydrodynamic radius was observed over this period, indicating good stability.

2.4 Elemental analysis of the allomelanin NPs and 1,8-DHN samples

Elemental analysis was conducted using a Thermo Scientific FLASH 2000 Series CHNS/O Elemental Analyzer (Thermo Fisher Scientific, Waltham, USA). The results for allomelanin NPs and the 1,8-DHN precursor are presented below:

Sample	%N	%C	%H	%O
**Allomelanin NPs**	0.163	69.02	4.10	26.72
**1,8-DHN**	0.000	74.90	4.99	20.11

Table S1: Elemental analysis of allomelanin and its precursor 1,8-DHN.

3. Transient absorption spectroscopy of allomelanin

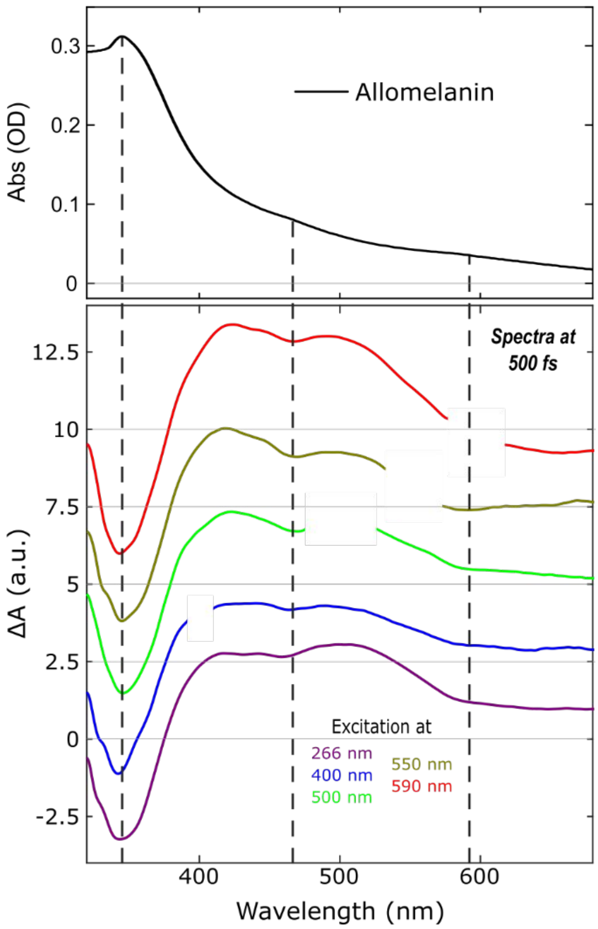

Figure S3: Absorption spectrum of the studied allomelanin (top panel), in comparison with the TA spectra obtained at 500 fs (bottom panel) upon excitation at 266 nm (violet), 400 nm (blue), 500 nm (green), 550 nm (gold), and 590 nm (red). The absorption peak at 350 nm appears as a ground state bleaching (GSB) signal in the TA datasets. Additionally, the TA spectra exhibit dips at 470 nm and 580 nm, positions that match the structured peaks of allomelanin’s absorption spectrum in the visible range, indicating that a broadband GSB may lie underneath.

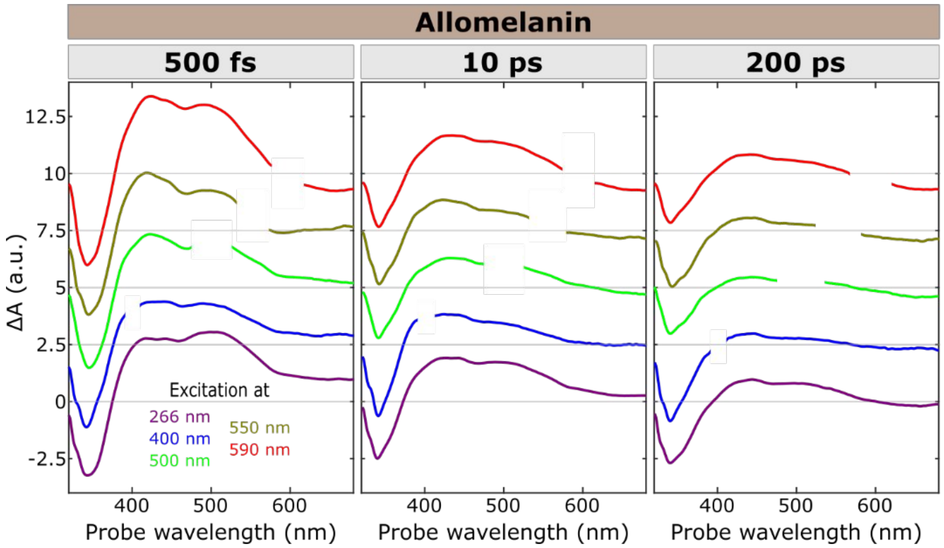

Figure S4: The TA spectra of allomelanin obtained at 500 fs (left panel), 10 ps (middle panel), and 200 ps (right panel) pump-probe delays, upon excitation at 266 nm (violet), 400 nm (blue), 500 nm (green), 550 nm (gold), and 590 nm (red). The data were acquired using tunable 100-fs pump pulses. The time delays were chosen to qualitatively match the “snapshots” of allomelanin's photoresponse at key temporal events: right after excitation (500 fs), after the sub-1 ps process of downhill energy transfer has been completed (10 ps), and after the formation of the photoproduct with a 24 ps time constant has been completed (200 ps).

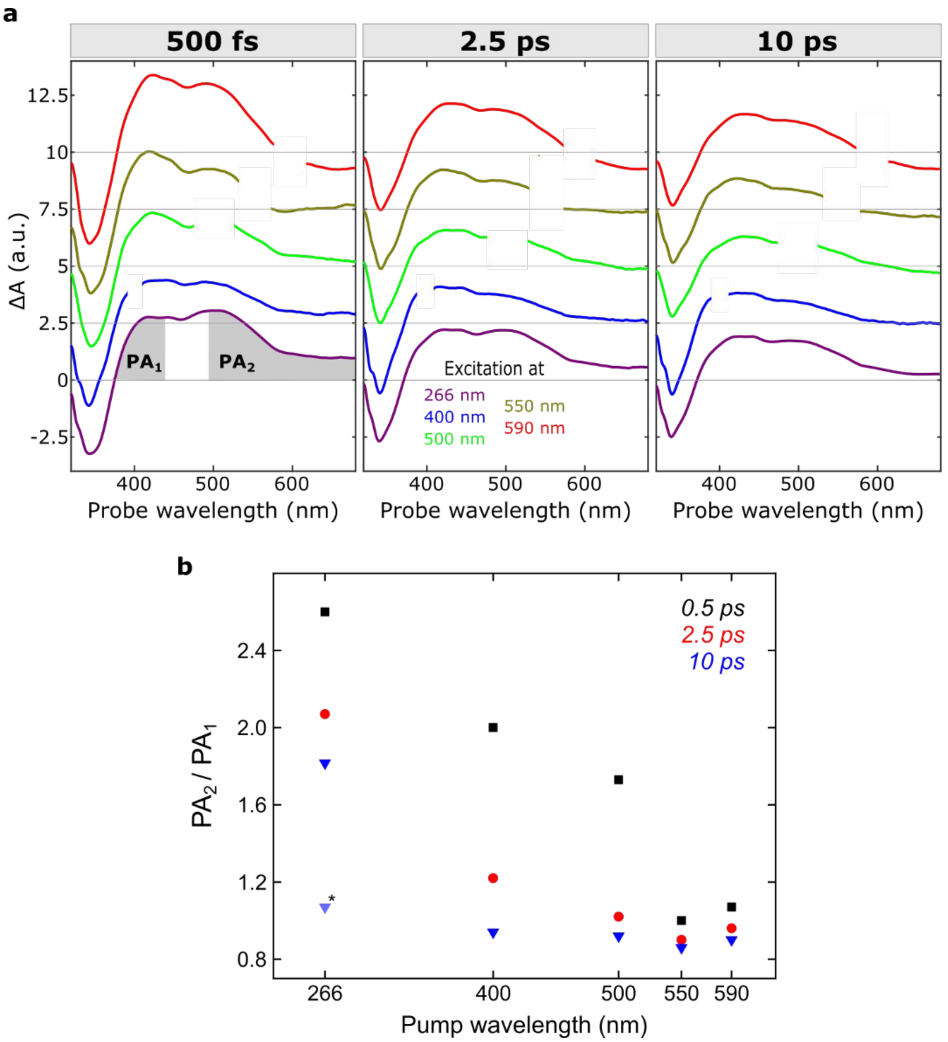

Figure S5: (a) The TA spectra of allomelanin obtained at 500 fs (left panel), 2.5 ps (middle panel), and 10 ps (right panel) pump-probe delay times, upon excitation at 266 nm (violet), 400 nm (blue), 500 nm (green), 550 nm (gold), and 590 nm (red). The PA_1 and PA_2 contributions, corresponding to the integration of the positive ΔA signals in the 375-440 nm and 490-675 nm regions, respectively, are highlighted in shaded grey colour. **(b)** The calculated ratio of PA_2/PA_1 as a function of the excitation wavelength, obtained for selected pump-probe delay times at 0.5 ps (black), 2.5 ps (red), and 10 ps (blue). At 0.5 ps, we observe a decrease in the PA_2/PA_1 ratio as the excitation wavelength increases. A similar trend, with gradually smaller magnitudes, is observed for longer delay times. For the individual excitation wavelengths, the ratio decreases sharply as a function of delay time for 266 nm, 400 nm, and 500 nm, while it shows minor decrease for 550 nm and 590 nm. The PA_2/PA_1 ratio for 266 nm excitation at 10 ps, calculated by fixing the PA_2 contributions at 490-560 nm to match the integrated region used for the other excitation wavelengths at 10 ps, is marked with a black asterisk. In case of using the same integrated windows for the PA_2 contribution, the PA_2/PA_1 ratios at 10 ps level off around 1±0.1, independent of the excitation wavelength.

3.1 Global analysis on fs-TA datasets

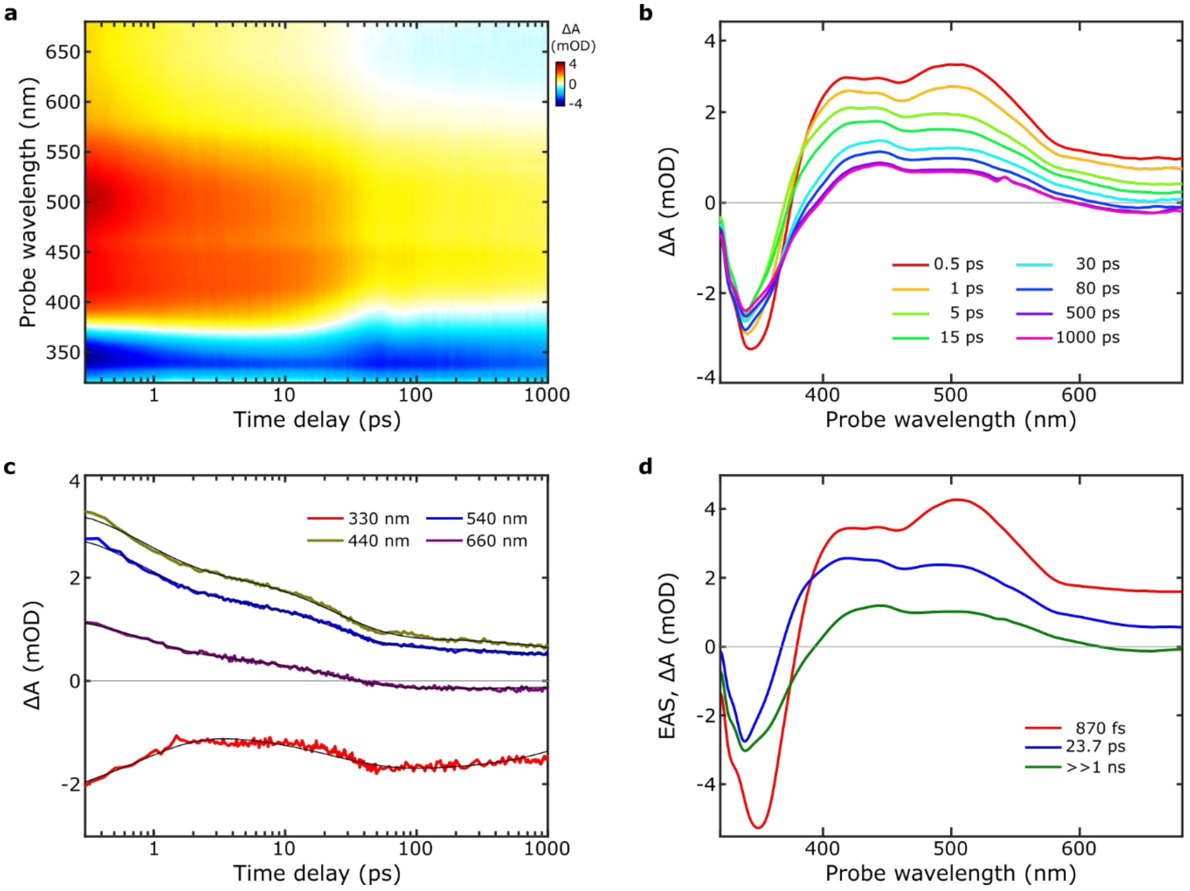

Figure S6: (a) TA map of allomelanin up to 1 ns delay times using 100-fs pump pulses centred at 266 nm (fs-TA). **(b)** TA spectra displayed for selected pump-probe delay times. **(c)** Dynamics at selected probe wavelengths, fitted using three exponential components, and **(d)** the evolution-associated spectra (EAS), accompanied by their corresponding time constants, retrieved by global analysis.

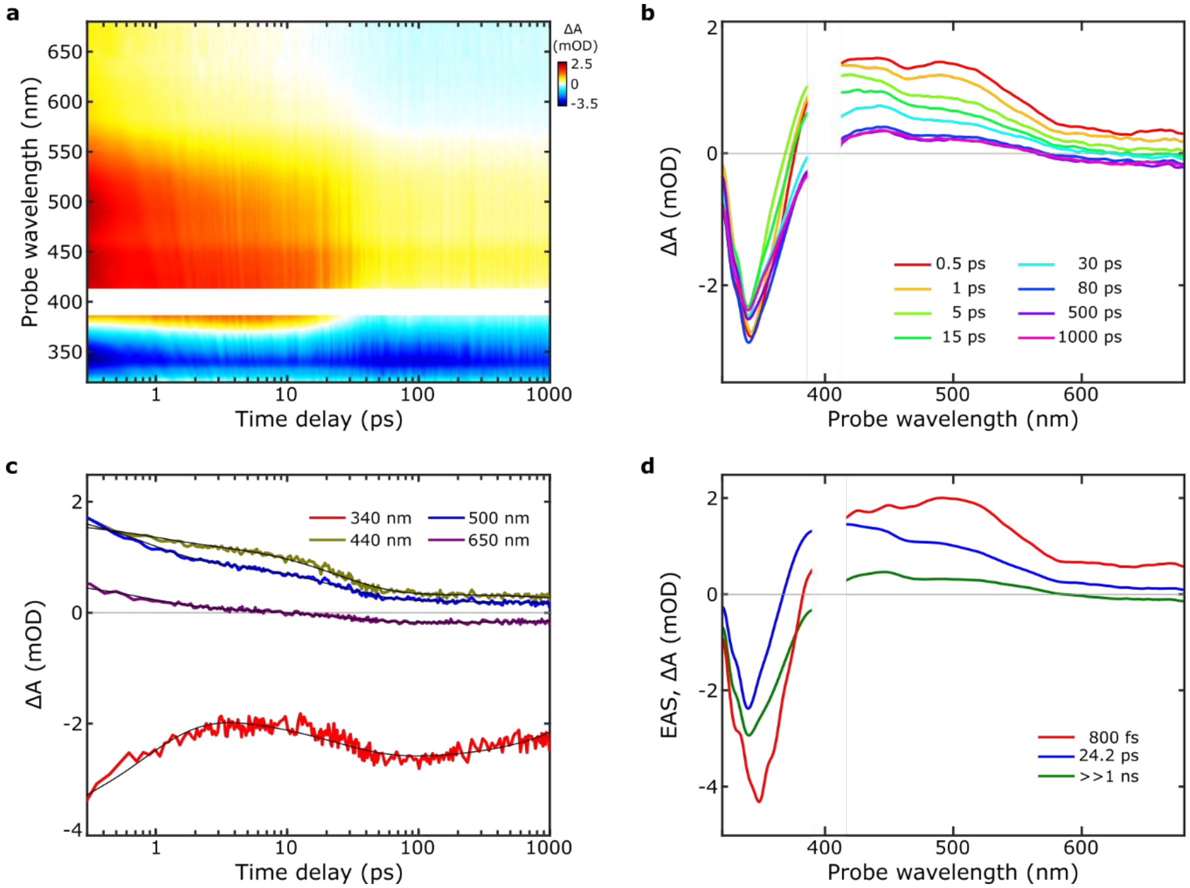

Figure S7: (a) Fs-TA map of allomelanin up to 1 ns delay times upon 400 nm excitation. **(b)** TA spectra displayed for selected pump-probe delay times. **(c)** Dynamics at selected probe wavelengths, fitted using three exponential components, and **(d)** the EAS, accompanied by their corresponding time constants, retrieved by global analysis. The probe region showing strong pump scattering has been omitted.

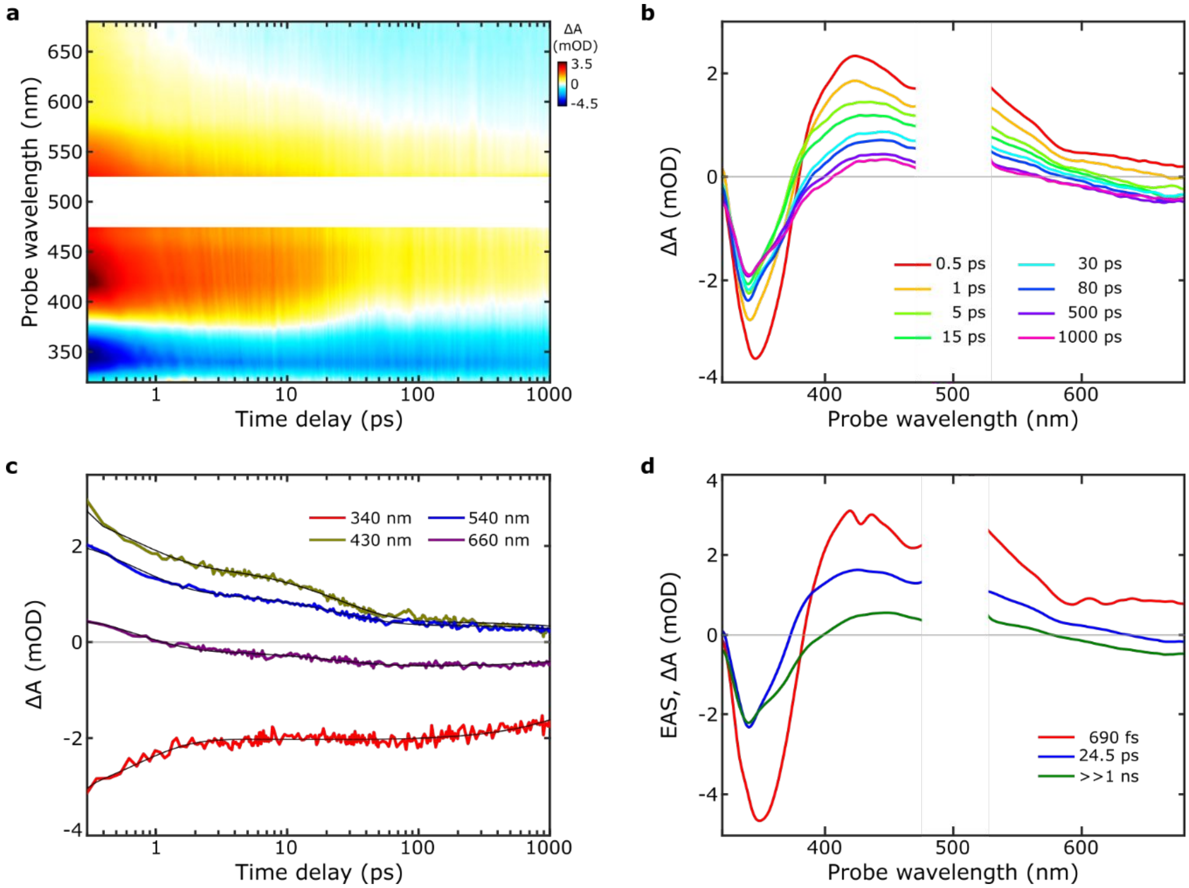

Figure S8: (a) Fs-TA map of allomelanin up to 1 ns delay times upon 500 nm excitation. **(b)** TA spectra displayed for selected pump-probe delay times. **(c)** Dynamics at selected probe wavelengths, fitted using three exponential components, and **(d)** the EAS, accompanied by their corresponding time constants, retrieved by global analysis. The probe region showing strong pump scattering has been omitted.

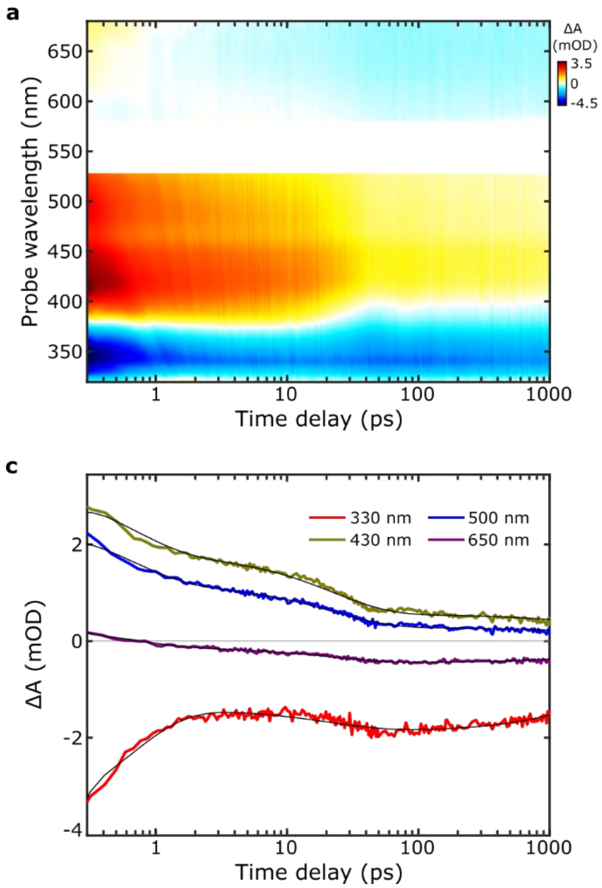

Figure S9: (a) Fs-TA map of allomelanin up to 1 ns delay times upon 550 nm excitation. **(b)** TA spectra displayed for selected pump-probe delay times. **(c)** Dynamics at selected probe wavelengths, fitted using three exponential components, and **(d)** the EAS, accompanied by their corresponding time constants, retrieved by global analysis. The probe region showing strong pump scattering has been omitted.

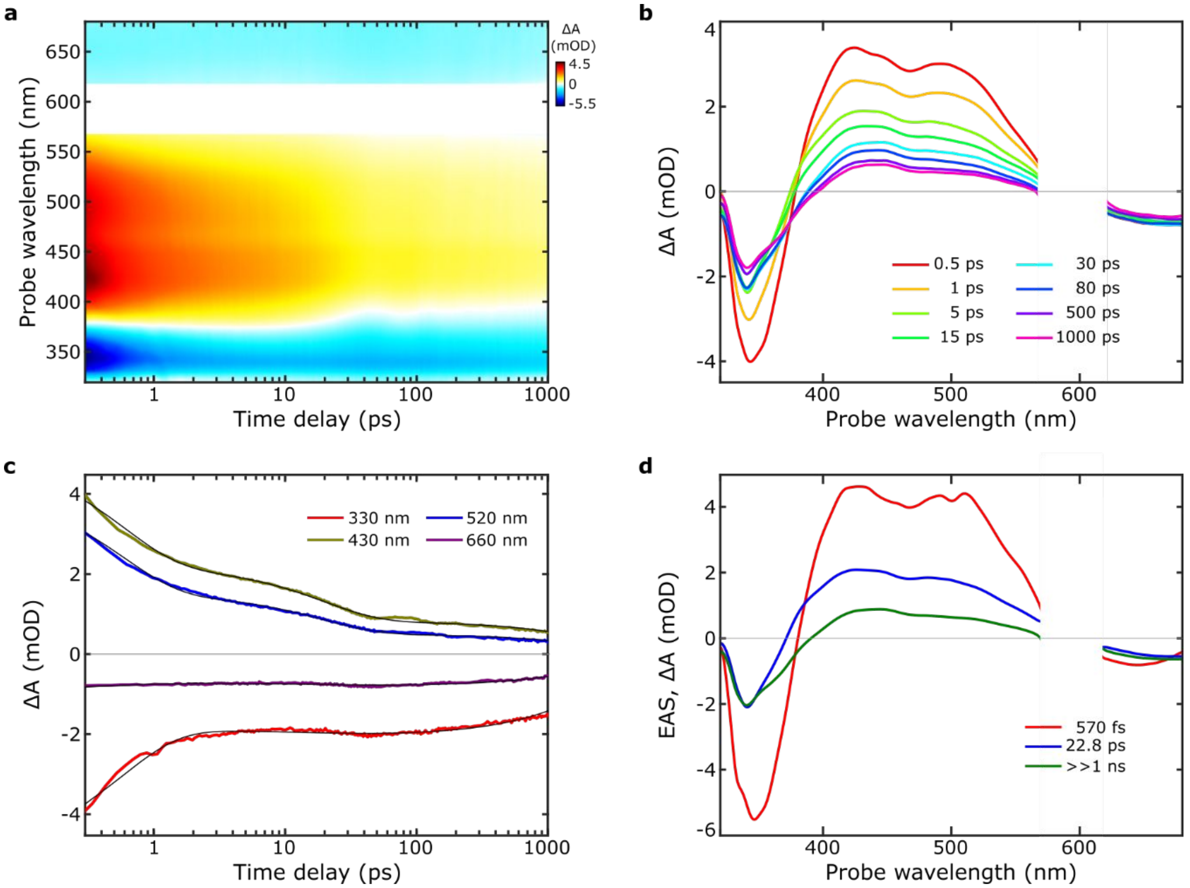

Figure S10: (a) Fs-TA map of allomelanin up to 1 ns delay times upon 590 nm excitation. **(b)** TA spectra displayed for selected pump-probe delay times. **(c)** Dynamics at selected probe wavelengths, fitted using three exponential components, and **(d)** the EAS, accompanied by their corresponding time constants, retrieved by global analysis. The probe region showing strong pump scattering has been omitted.

$\lambda_{excitation}$ (nm)	τ_1 (fs)	τ_2 (ps)	τ_3 (ns)

**266**	870	23.7	>> 1
**400**	800	24.2	>> 1
**500**	690	24.5	>> 1
**550**	640	23.2	>> 1
**590**	570	22.8	>> 1

Table S2: Summary of the TA fitting parameters, using three exponential components, extracted by global analysis for excitation wavelengths of 266 nm, 400 nm, 500 nm, 550 nm, and 590 nm, using fs-TA.

Excitation energy (eV)	τ_1 (fs)	k_{nr} x 10^{12} (s^{-1})	ln(k_{nr})
**4.66**	870	1.15	27.77
**3.10**	800	1.25	27.85
**2.48**	690	1.45	28.00
**2.25**	640	1.56	28.08
**2.10**	570	1.75	28.19

Table S3: Non-radiative rates (k_{nr}) of downhill energy transfer, corresponding to the τ_1 lifetimes obtained from global analysis at different excitation wavelengths/energies. As this process involves ultrafast relaxation and the overall emission quantum yield is negligible, radiative rates can be safely considered insignificant. At lower excitation energies, and thus for lower energy gaps, higher non-radiative rates (k_{nr}) are observed. However, the relationship between ln(k_{nr}) and energy gaps deviates from linearity, emphasizing the strongly coupled manifold of states involved.

3.2 Red-shift of the red-edge ground state bleaching signal

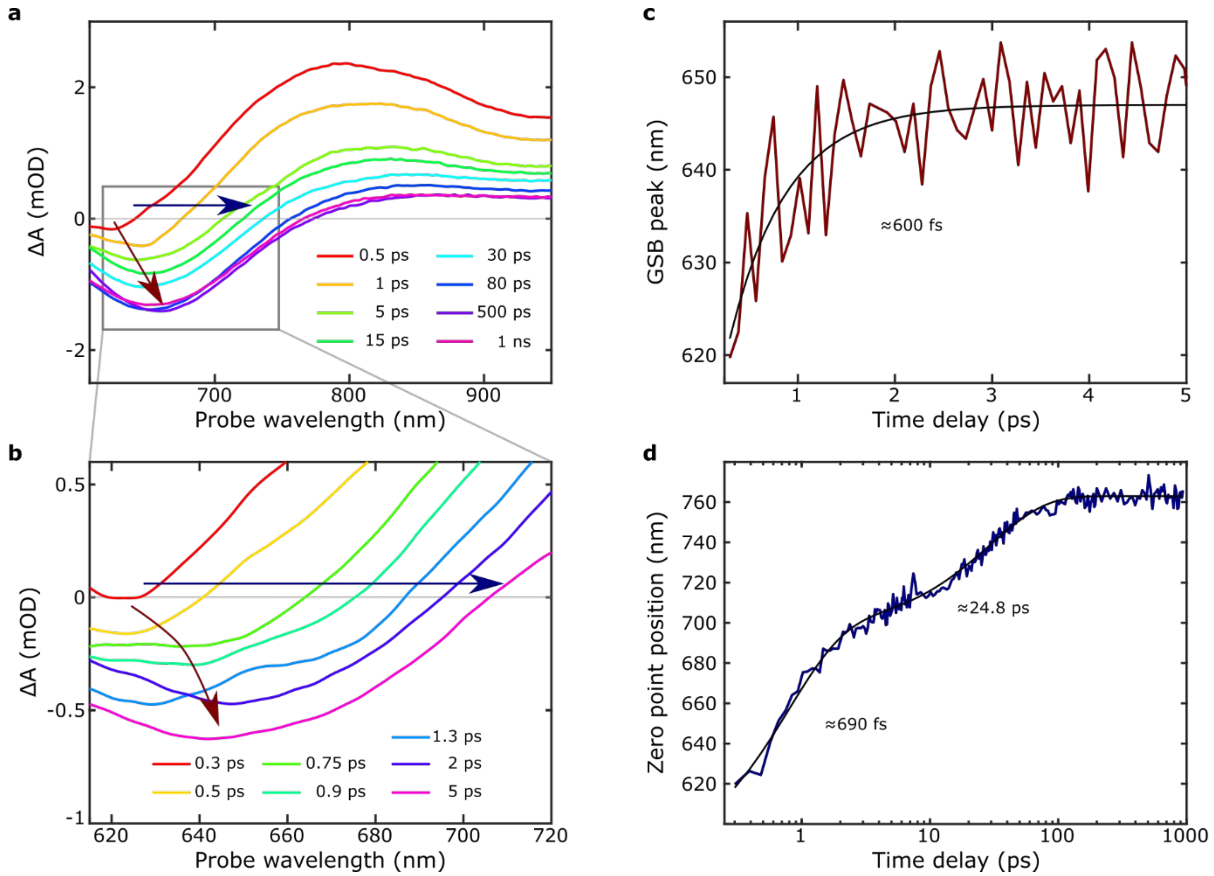

Figure S11: Fs-TA experiment for excitation at 550 nm with a probe window extending from 610-940 nm. **(a)** TA spectra for selected pump-probe delay times up to 1 ns. The grey coloured rectangle highlights the red-shifted signatures of both the GSB peak (brown arrow) and the probe wavelength corresponding to zero TA signal (zero position; dark blue arrow). The red-shift of the GSB peak is due to downhill energy transfer occurring on sub-1 ps timescales. The zero TA signal position results from the cancellation of the competing PA and GSB responses. Consequently, the zero position is influenced initially by the red-shift of the broad GSB peak and by the subsequent decay of PA without recovery of the GSB, due to the formation of terminal photoproduct species within approximately 24 ps. **(b)** A closer look at the 610-720 nm detection range shows the spectral evolution within the first 5 ps, with the GSB peak and zero position shifts highlighted by brown and dark blue arrows, respectively. **(c)** The GSB peak appears at 620 nm immediately after photoexcitation and, after an ultrafast shift of 600 fs, levels off at 647 nm. The broad GSB bands and the small (< 30 nm) shift of the GSB peak have as a result a peak shift that is sensitive to the experimental noise of the individual dynamics of close lying detection wavelengths. Conversely, the zero position is influenced less by the noise due to its large shift from 620 nm to 763 nm. **(d)** The zero position shift manifests the energy transfer event (690 fs) and the subsequent PA decay towards the formation of photoproducts (24.8 ps), eventually stabilizing around the 763 nm probe wavelength at later timescales.

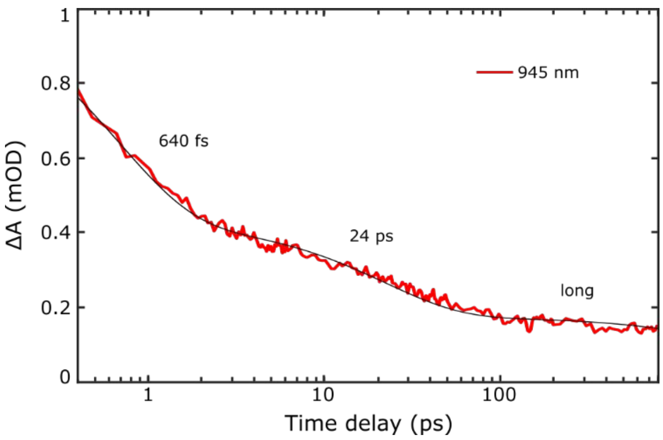

Figure S12: Kinetics of allomelanin NPs in water following 550 nm excitation, with detection at 945 nm in the NIR region. The probe kinetics are measured at long wavelengths where the GSB signal contribution is negligible. Nonetheless, for accurate fitting the decay needs three-exponential terms.

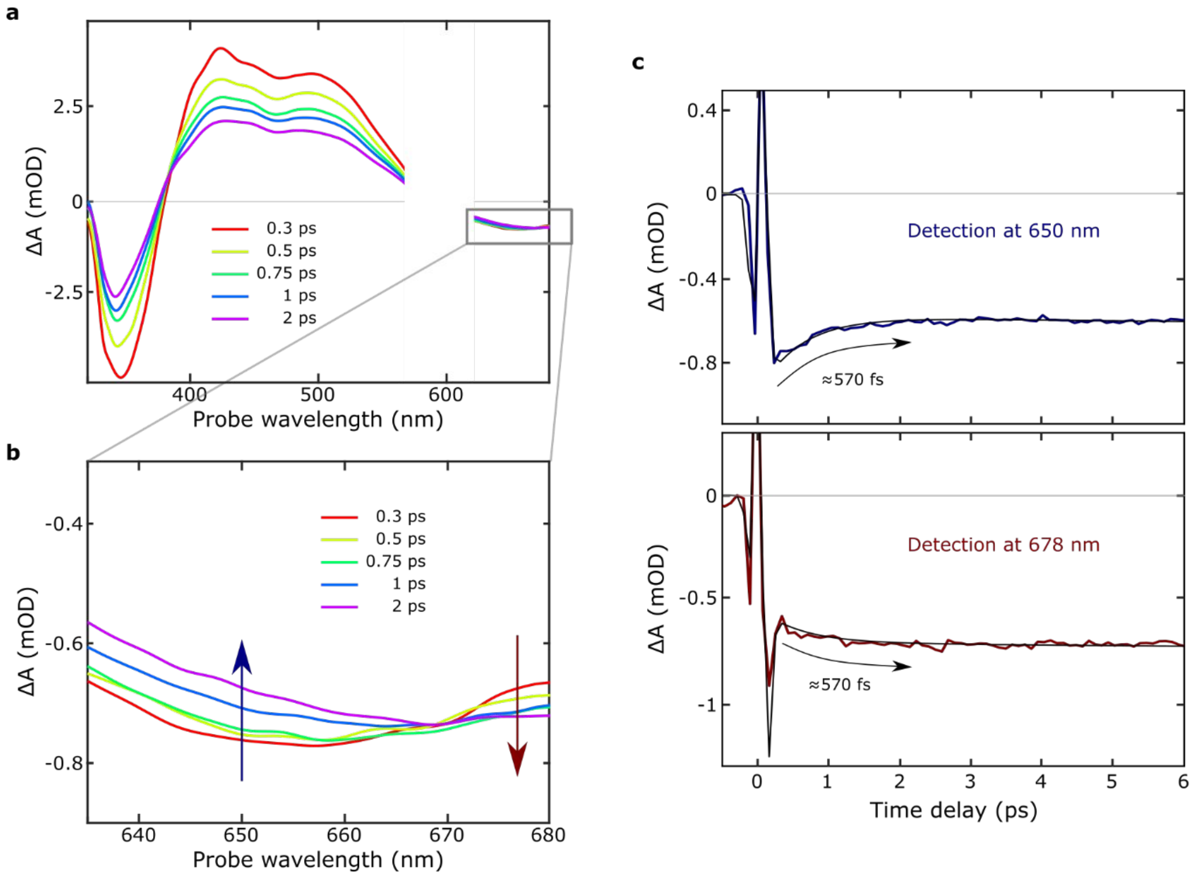

Figure S13: Fs-TA experiment for excitation at 590 nm , focusing on the sub-2 ps spectral evolution. TA spectra are shown for selected pump-probe delay times up to 2 ps, **(a)** for the 320-

680 nm probe range and **(b)** focusing on the 630-680 nm probe range. On the sub-2 ps timescale, there is an evident shift of the GSB peak from 650 nm to longer wavelengths, as indicated by the arrows highlighting the evolution at 650 nm (dark blue) and 678 nm (brown). **(c)** The decay of the 650 nm probe wavelength matches **(d)** the rise time of the 678 nm wavelength, with an approximate lifetime of 570 fs, as extracted by the global fitting shown in Figure S10, highlighting the red-shift of the GSB due to the downhill energy transfer. The red-shift is further highlighted transitioning from EAS_1 to EAS_2 in Figure S10.

3.3 Global analysis on ns-TA datasets

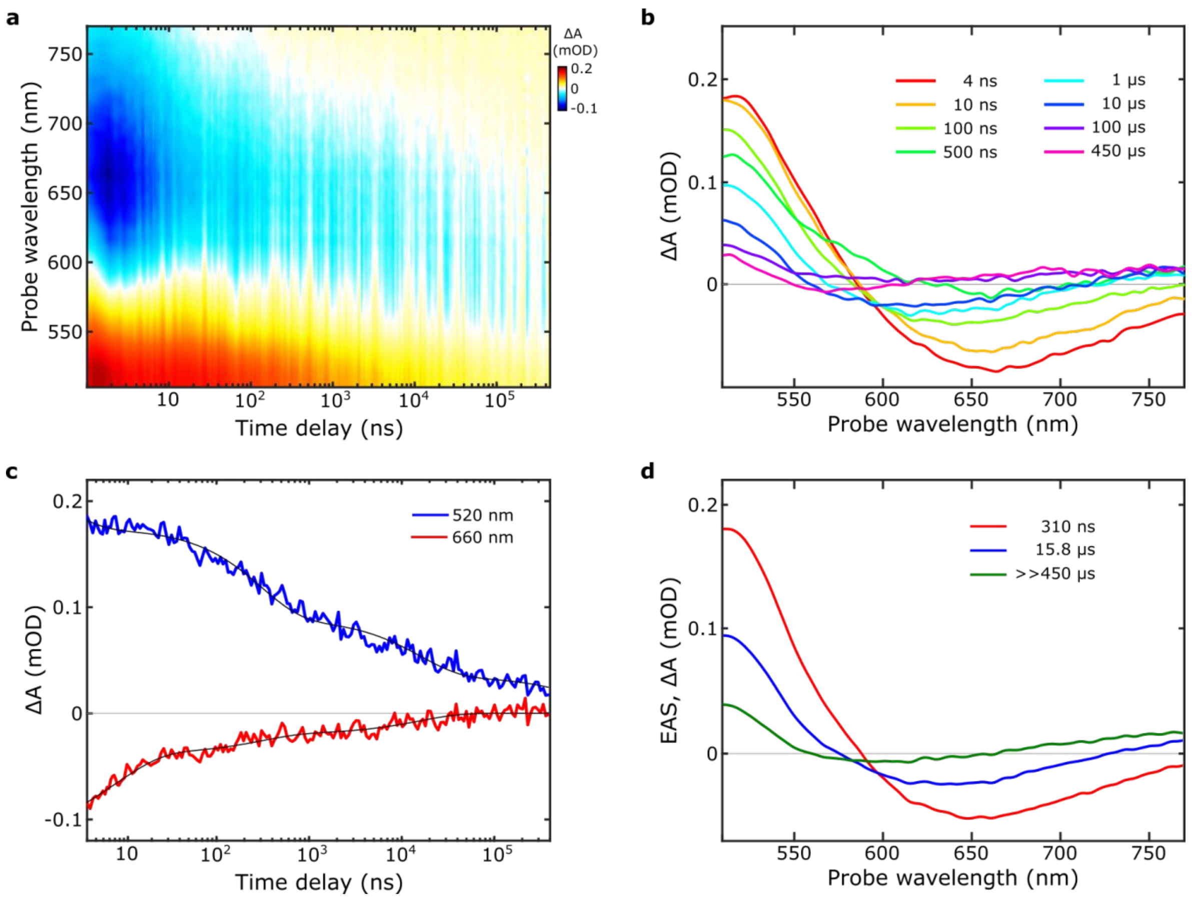

Figure S14: (a) Ns-TA map of allomelanin up to 450 μs delay times using 355 nm pump pulses with an approximately 800 ps width. **(b)** TA spectra for selected pump-probe delay times ranging from 4 ns to 450 μs. **(c)** Dynamics at selected probe wavelengths, approximated by three-exponential fitting, and **(d)** the EAS with their corresponding time constants, retrieved by global analysis.

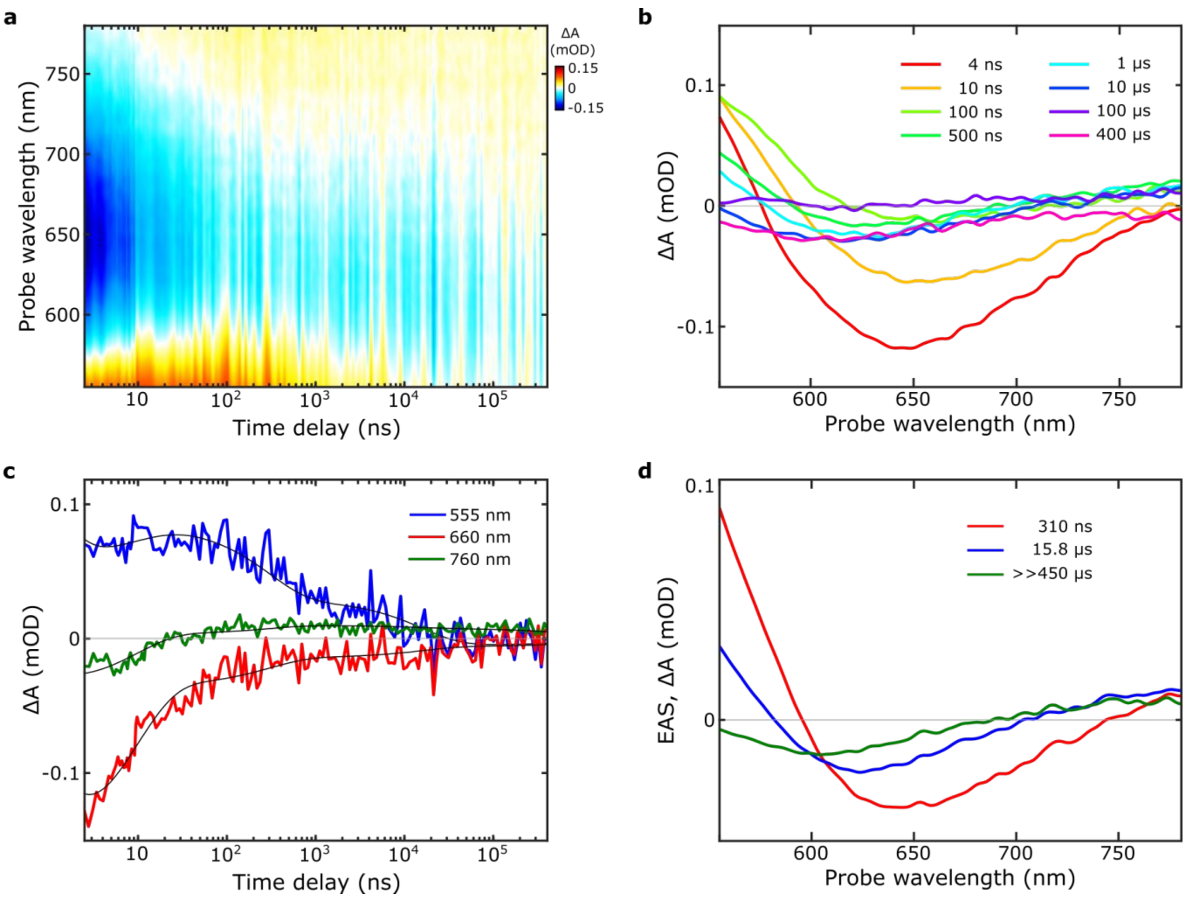

Figure S15: (a) Ns-TA map of allomelanin NPs in water up to a 450 μs delay using 532 nm pump pulses with an approximate width of 800 ps. **(b)** TA spectra at selected pump-probe delays from 4 ns to 400 μs. **(c)** Dynamics at specific probe wavelengths, approximated by three-exponential fitting. **(d)** EAS and their corresponding time constants, obtained via global analysis. The fitted lifetimes were kept fixed in this experiment, in order to have a direct comparison of their weighted amplitudes with respect to the 355 nm excitation experiment.

$\lambda_{excitation}$ (nm)	A_1 (%)	τ_1 (ns)	A_2 (%)	τ_2 (μs)	A_3 (%)	τ_3 (μs)	$<\tau_{rec}>^a$ (μs)
**355**	50	310	34	15.8	16	>> 450	6.6
**532**	55	310	37	15.8	8	>> 450	6.7

[a] The average recombination lifetime was calculated as a weighted sum of the first two decay components, which clearly decay within our temporal window and are associated with recombination pathways. The third, non-decaying component, observed as a plateau in our measurements, is attributed to long-lived stabilized free radicals.

Table S4: Fitting parameters extracted through global analysis for excitations at 355 nm and 532 nm using ns-TA reveal that the main recombination decay lifetimes are comparable for both

excitations. However, the long-lived (>> 450 μs) residual component exhibits twice the amplitude under 355 nm excitation.

4. Fluence dependent transient absorption measurements

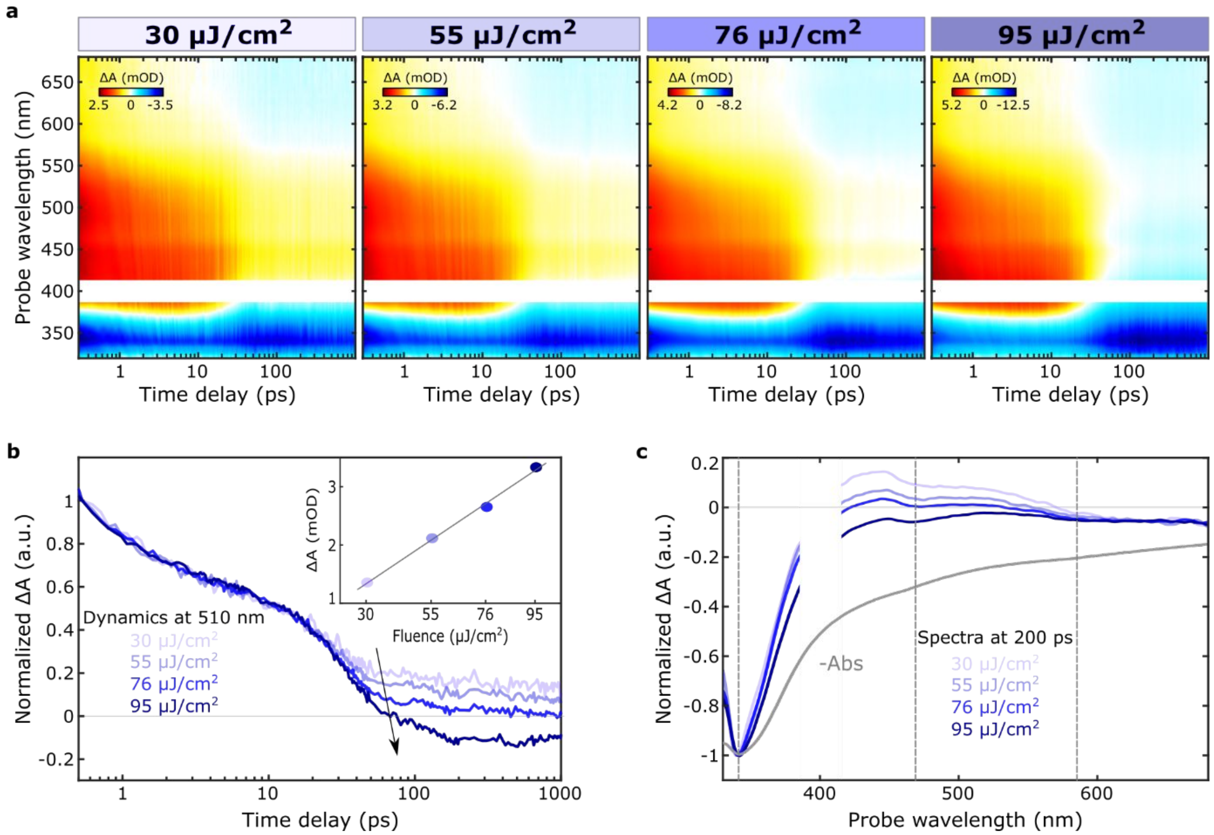

Figure S16: Fluence-dependent measurements of allomelanin for excitation at 400 nm, using fs-TA. **(a)** TA maps up to 1 ns delay times for excitation fluences of 30 μJ/cm², 55 μJ/cm², 76 μJ/cm², and 95 μJ/cm² (from left to right panels). **(b)** Normalized TA dynamics at 510 nm, showing variation with excitation fluence. Colours range from light to dark blue, corresponding to low to high excitation fluence, respectively. **(c)** Normalized TA spectra at 200 ps for different fluences, with the absorption spectrum of allomelanin superimposed to identify similar peaks in the TA spectra observed as GSB. The vertical dotted lines at 350 nm, 470 nm, and 580 nm highlight the common signatures.

To delve deeper into the character of the excited states, we conducted fluence-dependent measurements following 400 nm excitation (Figure S16a,b). As shown in the inset of Figure S16b, the initial amplitude of the PA signal at 510 nm linearly increases as a function of exciton density, ruling out excitonic interactions from different absorption events within the pump pulse duration. Similarly, the two-step mechanism involving exciton migration and decay to the terminal photoproduct state appears to be nearly pump-fluence independent, with the non-linearity of the PA signal primarily arising from the population of the photoproduct (Figure S16b). In transient spectroscopic studies, a key signature of exciton mobility is the predominant exciton-exciton annihilation at high exciton densities.[8,9] Mobile excitons from different absorption events may collide and exchange energy, resulting in bimolecular interactions that quench the excited state population reducing its lifetime, with the probability of such events increasing as the excitation fluence rises.

Additionally, the annihilated PA at high fluences reveals the broadband GSB lying underneath, closely matching the broadband allomelanin absorption spectrum (Figure S16c). This result further supports the presence of broadband-coupled electronic transitions from UV to NIR energies and the competition of PA and GSB signals within our probe window. Moreover, the alignment of the negative ΔA peaks, attributed to GSB due to the low emission quantum yields in the visible, with the sub-peaks of the absorption spectrum in the visible range, further indicates that stimulated emission (SE) is negligible. Efficient SE would manifest as a shift in the negative ΔA signal or the appearance of new peaks.

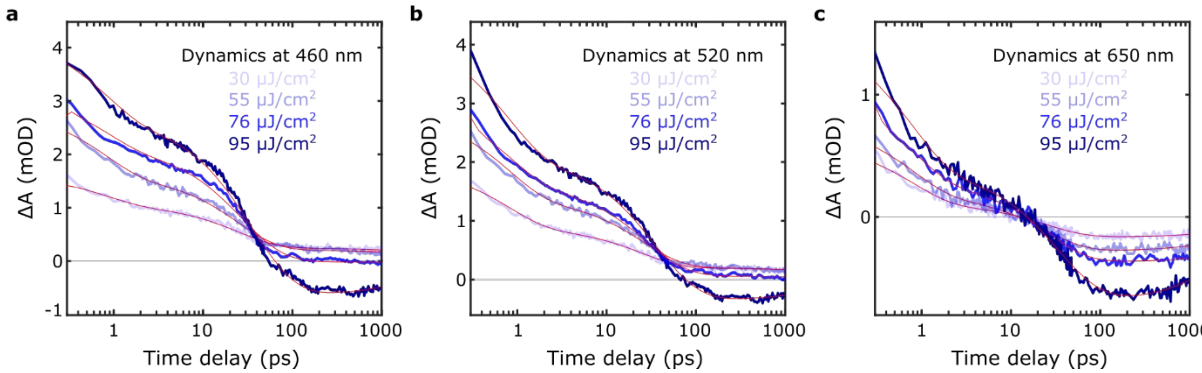

Figure S17: Dynamics (light blue to dark blue lines) and their fitting curves (red lines) extracted by global fitting for different excitation fluences. Four exponential components were used (800 fs, 24.2 ps, 46 ps, >> 1 ns) with varying amplitudes. Detection wavelengths are **(a)** 460 nm, **(b)** 520 nm, and **(c)** 650 nm.

As shown in Figure S7 for 400 nm excitation, the low fluence TA measurements are satisfactorily fitted with three exponential components (800 fs, 24.2 ps, >>1 ns). In contrast, the dynamics obtained for the maximum used fluence at 400 nm clearly exhibit an additional component on sub-300 ps timescales (Figure S16b and Figure S17). Indeed, four exponential components (800 fs, 24.2 ps, 46 ps, >>1 ns) are necessary to fit the high fluence TA datasets. To evaluate the amplitude weight of the additional 46 ps component as a function of the excitation fluence, we kept the lifetime components from the global fits of the high fluence measurements fixed while globally fitting our datasets. The varying amplitudes are shown in Table S5.

We categorize the lifetimes and their corresponding amplitudes into two groups: Frenkel exciton species, which predominate immediately after photoexcitation and during exciton migration in sub-10 ps timescales, and photoprodcut species of different character formed at later timescales. The quenching of the excited state dynamics with increasing exciton density primarily arises from the presence of the 46 ps component, which becomes predominant at high fluences (44% of the two long lived components). We note that the datasets obtained at the lowest excitation fluence of 400 nm have a minor contribution from the 46 ps lifetime component (2% of the two long lived components). This is expected, as the dynamics can be well-fitted by three exponentials. This three-exponential behavior is generally observed across different excitation wavelengths obtained under the low fluence regime. Thus, the presence of three exponentials at different excitation wavelengths can be used as an indicator that the TA datasets are obtained within the low fluence linear regime.

	**Frenkel exciton specie**				**Photoproduct**			
**Fluence at 400 nm**	**A_1 (%)**	**τ_1 (fs)**	**A_2 (%)**	**τ_2 (ps)**	**A_1 (%)**	**τ_1 (ps)**	**A_2 (%)**	**τ_2 (ns)**
**30 μJ/cm²**	55	800	45	24.2	2	46	98	>> 1
**55 μJ/cm²**	53	800	47	24.2	8	46	92	>> 1
**76 μJ/cm²**	49	800	51	24.2	21	46	79	>> 1
**95 μJ/cm²**	52	800	48	24.2	44	46	56	>> 1

Table S5: Fitting parameters extracted by global analysis for excitation at 400 nm with varying pump fluence. Four exponential components were identified by fitting the high-fluence datasets and were kept fixed for the lower fluences to obtain the varying amplitudes corresponding to each lifetime component.

As shown in Table S5, the Frenkel excitonic lifetimes and amplitudes are largely unaffected by increasing exciton density. While the short-lived Frenkel excitons in allomelanin may exhibit some mobility, no significant excitonic interactions are observed within the range of explored fluences.

We focus on the transition to low-energy photoproduct states, which form independently of the excitation wavelength and coincide with the onset of excited-state annihilation. This transition, from EAS_2 to EAS_3, is supported by the absence of GSB recovery alongside a significant decay in the PA population. However, the lack of new PA signals leaves the evidence for new species formation indicative but inconclusive. Fluence-dependent measurements provide further support for the emergence of new species beyond approximately 24 ps.

The non-linear behavior of transient signals after 24 ps suggests that these non-emitting photoproduct species act as Frenkel excitonic traps. This non-linearity may arise from either increased mobility of the photoproduct species or a higher degree of unbound excitonic strength in the excited state compared to the initial bound excitonic species.[9]

5. Measurements on allomelanin NPs dispersed in thin film, water and organic solvents

In this section, we present measurements of allomelanin nanoparticles (NPs) dispersed in thin films, water, acetonitrile, methanol, and ethanol. Our focus is on the effects of

molecular packing and solvent environment on their absorption spectral features and excited state kinetics. The predominant excited state character is proposed to be of excitonic (before the 24 ps lifetime) and subsequently of charge transfer (after the 24 ps lifetime) in origin (see “Fluence dependent transient absorption measurements” section). As such, it is of particular interest the effect of the molecular packing and solvent polarity on their excited state lifetimes.

In organic solvents, allomelanin NPs tend to disaggregate into smaller species, consistent with prior studies.[10] This is evident in Figure S18, where NPs in organic solvents display the vibronic pattern of the 1,8-DHN precursor in the UV range, which is absent in thin films and aqueous dispersions, along with an extended absorption tail from the visible to the NIR range. These observations highlight an equilibrium between monomeric species and higher degree of aggregates in organic solvents. Notably, the absorption spectra of de-aggregated NPs lack the characteristic 470 nm peak, suggesting this feature is size-dependent.[10] In contrast, the 580 nm peak, present in all conditions, is size-independent and attributed to excitonic features of conjugated quinoid oligomers.[11,12] DLS measurements confirm the presence of NPs in organic solvents; however, a significant decrease in scattering intensity compared to NPs in aqueous solution indicates partial disaggregation. This finding is further supported by TEM images reported in a previous publication.[10]

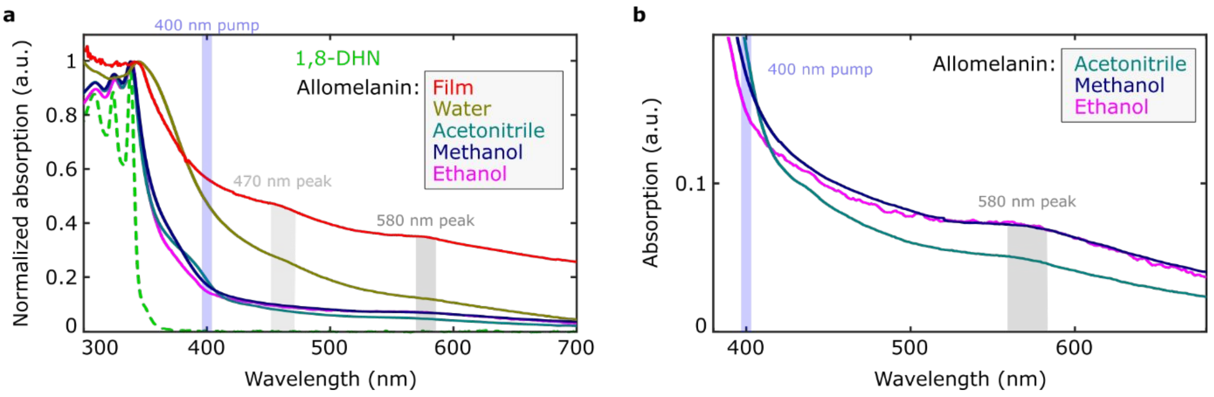

Figure S18: (a) Absorption spectra of allomelanin NPs dispersed in a thin film (red solid line), water (gold solid line), acetonitrile (teal solid line), methanol (blue solid line), and ethanol (magenta solid line), compared to the reference absorption spectrum of the 1,8-DHN allomelanin precursor (green dotted line). The blue-shaded rectangle indicates the excitation wavelength at 400 nm used for the transient absorption experiments discussed in this section, where absorption from potential monomeric contributions is negligible. The gray-shaded rectangles highlight the absorption shoulders at 470 nm and 580 nm. **(b)** A zoomed-in view of the absorption spectra for allomelanin NPs in organic solvents (acetonitrile, methanol, and ethanol), where the nanoparticles dissociate into smaller aggregates. The 580 nm absorption peak remains present, but the shoulder at 470 nm is absent, in stark contrast to the nanoparticles in film and water, where both peaks are pronounced.

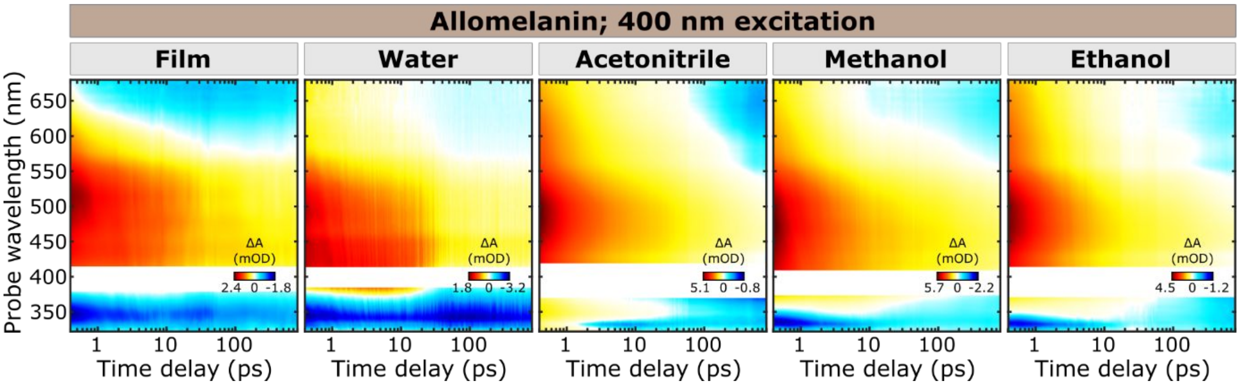

Figure S19: Fs-TA maps of allomelanin NPs as a function of probe wavelength and time, following 400 nm excitation. The NPs are dispersed in a thin film, water, acetonitrile, methanol, and ethanol, respectively (from left to right).

In thin films, where NPs are densely packed, the absorption spectrum closely resembles that of aqueous dispersions but exhibits a stronger visible-NIR component (Figure S18). This enhancement likely reflects stronger intermolecular interactions in the film due to denser packing. Excitation at 400 nm primarily targets smaller aggregated species in organic solvents, as the monomeric 1,8-DHN precursor does not absorb significantly at this wavelength. Conversely, aqueous dispersions and thin films predominantly involve larger aggregates, with particles around 170 nm in diameter (see DLS results) acting as the absorbing population.

Despite differences in NP size and solvent environment, all samples exhibit qualitatively similar spectral signatures and temporal evolution after 400 nm excitation (Figure S19). Notably, no spectral hole burning is observed, even in de-aggregated samples, indicating that smaller NPs retain broad excitonic couplings and exhibit similar kinetic behavior. This robustness underscores the intrinsic properties of the allomelanin system.

Global fitting of the transient absorption datasets reveals that aqueous NPs excited at 400 nm require three exponential components (800 fs, 24.2 ps, >1 ns) to describe their dynamics (the fitting was shown in Figure S7). In organic solvents, the coexistence of various de-aggregated species leads to more complex multi-exponential decays, approximated by four components in acetonitrile (Figure S21), ethanol (Figure S22), and methanol (Figure S23). The first two EAS components in organic solvents resemble the blue-shifted PA features, associated with the 800 fs lifetime found in water, while the third component corresponds to the PA decay without GSB recovery, characteristic of the 24 ps lifetime found in water. Overall, the processes in organic solvents appear similar but exhibit slower average kinetics, likely due to smaller particle size and weaker packing.

Thin-film measurements reveal a three-exponential decay similar to aqueous dispersions, but all kinetic processes occur significantly faster (Figure S20; Table S6). This acceleration is attributed to the denser packing of NPs in the film, which enhances

intermolecular interactions and excitonic coupling. Importantly, no clear correlation is observed between solvent polarity or protic activity and the observed dynamics in organic solvents (Table S6), in where NPs size can be comparable. The overall kinetic behavior suggests that NP size and molecular packing dominate over the solvent environment in determining both spectral and kinetic behavior.

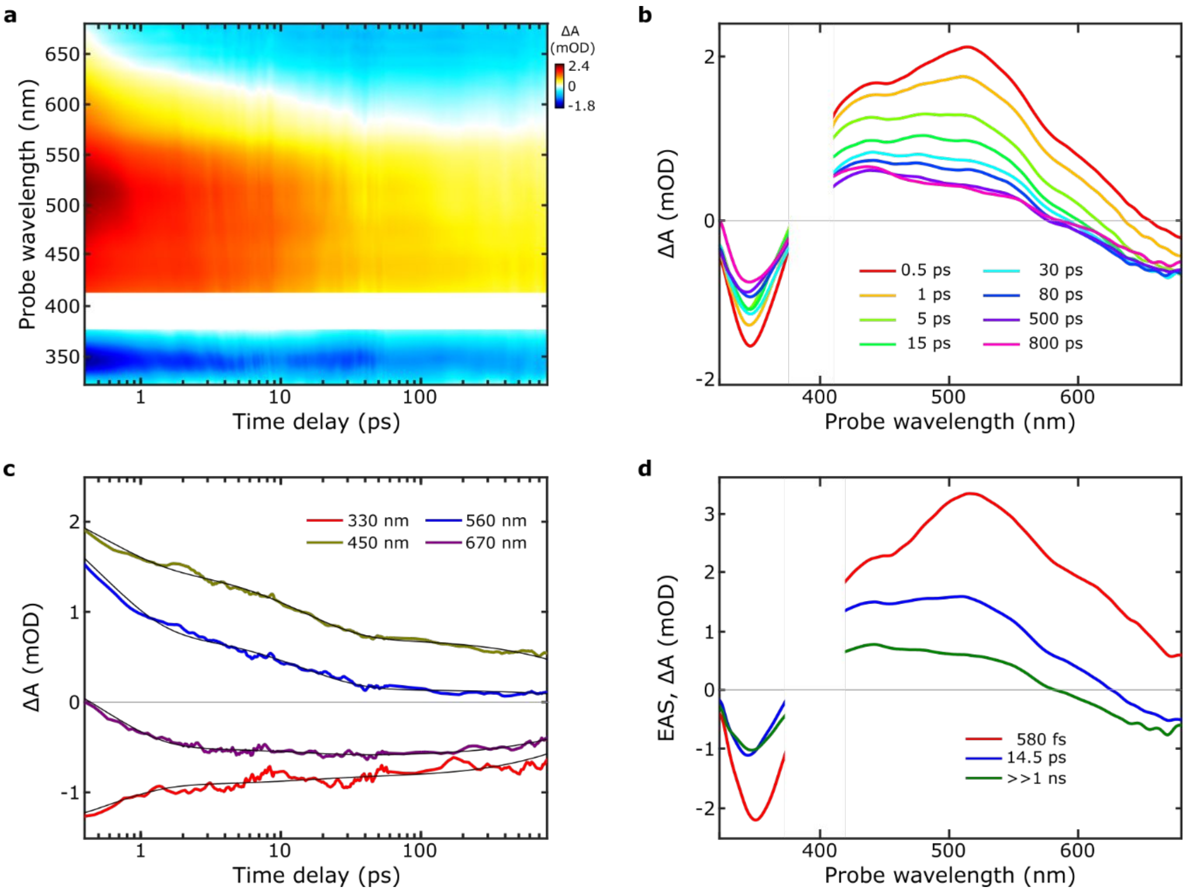

Figure S20: (a) Fs-TA map of allomelanin NPs deposited on a thin film, recorded up to 1 ns delay following 400 nm excitation. **(b)** TA spectra at selected pump-probe delay times. **(c)** Dynamics at specific probe wavelengths, fitted using three exponential components. **(d)** EAS and their corresponding time constants, obtained through global analysis. Regions affected by strong pump scattering have been omitted.

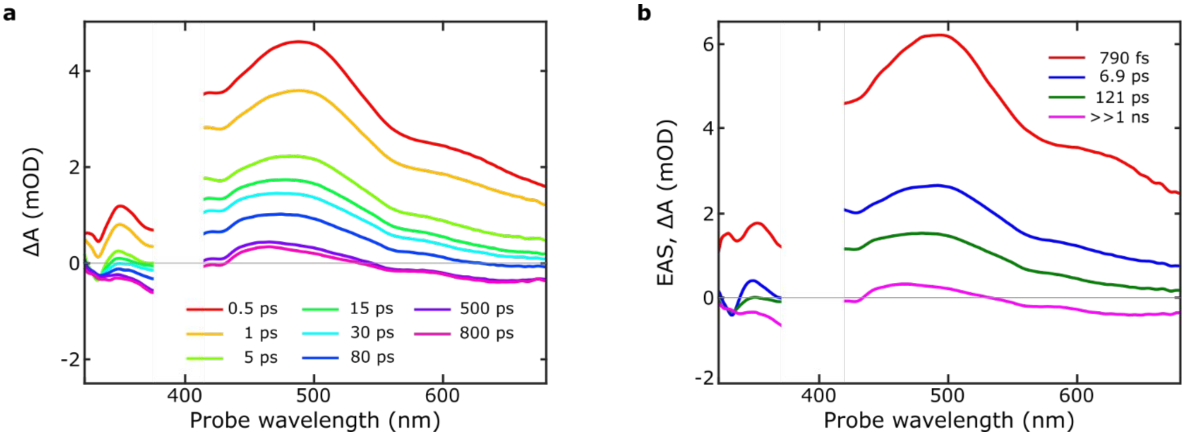

Figure S21: (a) Fs-TA spectra of allomelanin NPs dispersed in acetonitrile at selected pump-probe delay times up to 1 ns following 400 nm excitation. Regions affected by strong pump scattering have been omitted. **(b)** EAS components with their corresponding time constants, obtained through global fitting of the presented datasets.

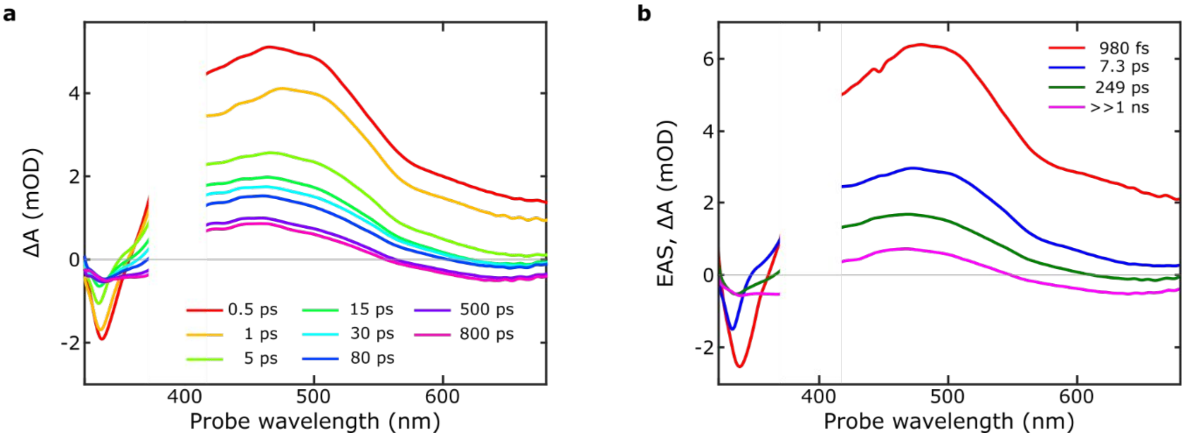

Figure S22: (a) Fs-TA spectra of allomelanin NPs dispersed in methanol at selected pump-probe delay times up to 1 ns following 400 nm excitation. Regions affected by strong pump scattering have been omitted. **(b)** EAS components with their corresponding time constants, obtained through global fitting of the presented datasets.

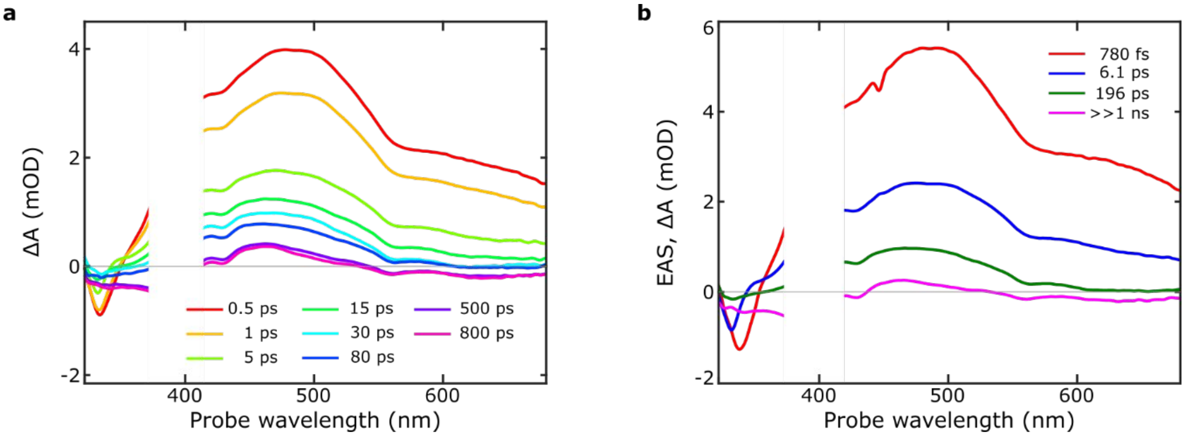

Figure S23: (a) Fs-TA spectra of allomelanin NPs dispersed in ethanol at selected pump-probe delay times up to 1 ns following 400 nm excitation. Regions affected by strong pump scattering have been omitted. **(b)** EAS components with their corresponding time constants, obtained through global fitting of the presented datasets.

Sample	τ_1 (fs)	τ_2 (ps)	τ_3 (ps)	τ_4 (ns)
**Film**	580	14.5		>> 1
**Water**	800	24.2		>> 1
**Acetonitrile**	790	6.9	121	>> 1
**Methanol**	980	7.3	249	>> 1
**Ethanol**	780	6.1	196	>> 1

Table S6: Fitting parameters obtained from global analysis of fs-TA data for 400 nm excitation of allomelanin NPs in a thin film, water, acetonitrile, methanol, and ethanol. A three-exponential model is required for the thin film and water environments, whereas a four-exponential model is necessary for the organic solvents.

6. Reversible changes in the absorption of allomelanin NPs upon irradiation

To demonstrate the accumulation of a long-lived stabilized species upon light irradiation, we performed a time-resolved absorption experiment on the minute timescale. The absorbance at 850 nm of an allomelanin NP suspension was monitored using an Agilent 8453 spectrophotometer equipped with a diode array detector. As shown in Figure S24, an LED (400 nm, OSRAM) was focused on the NP suspension, and the absorbance at 850 nm was tracked for 600 s. After this period, the light was switched off. The use of diode array detectors was necessary to prevent interference from the LED light.

As shown in Figure S24, an exponential increase in absorbance at 850 nm, with a rate constant of $k = 0.023 \pm 0.002\ s^{-1}$, was observed during light irradiation. Upon switching off the LED, the signal exhibited an exponential decay, demonstrating fully reversible absorption changes, with a rate constant of $k = 0.016 \pm 0.001\ s^{-1}$.

As discussed in detail in the main text, these reversible absorption changes correspond to photoproducts with a predominantly anionic semiquinone-like radical character.

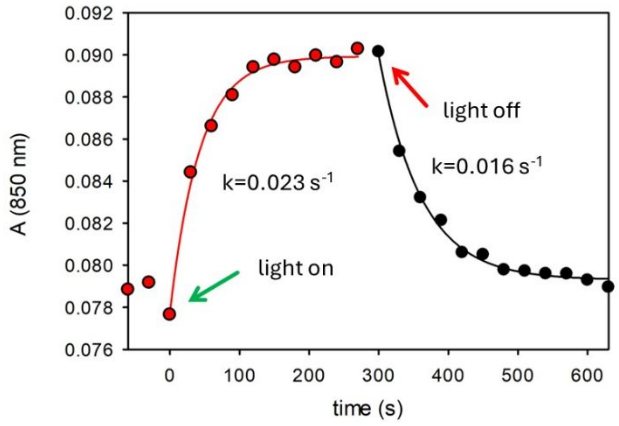

Figure S24: Absorbance at 850 nm of allomelanin NP in water during irradiation with LED at 400 nm. Light was switched on a time 0 s for 300 s and then switched-off

7. Investigation of intermediate triplet states in allomelanin

The potential quenching of $[Ru(bpy)_3]^{2+}$, a metal complex known to undergo intersystem crossing to its triplet excited state with nearly 100% efficiency, by allomelanin NPs was investigated. Specifically, the excited-state decay of a 0.3 mM aqueous solution of the complex was recorded using a Time-Correlated Single Photon Counting (TCSPC) system (Edinburgh FLS 1000). Luminescence decay measurements were first performed after bubbling Ar for 15 minutes, then repeated in the presence of allomelanin NPs (0.1 mg/mL).

As shown in Figure S25, no significant difference was observed in the luminescence decay, demonstrating that allomelanin does not quench the triplet excited state of $[Ru(bpy)_3]^{2+}$ under these experimental conditions. This effectively rules out triplet-triplet energy transfer from $[Ru(bpy)_3]^{2+}$ to possible allomelanin triplet intermediates.

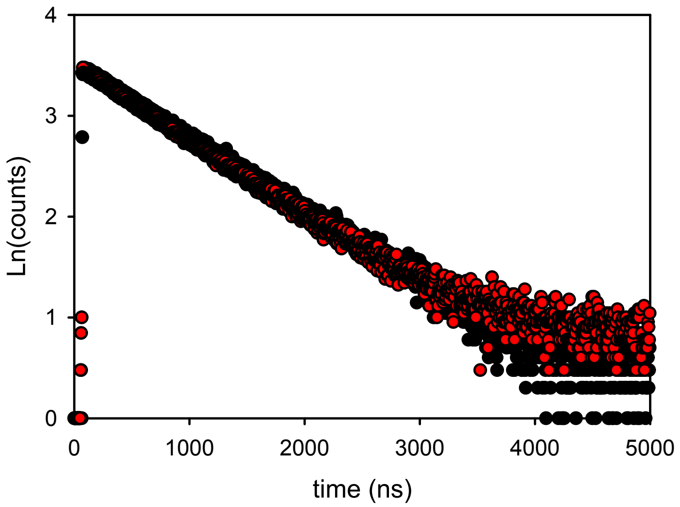

Figure S25: Excited-state decay of $[Ru(bpy)_3]^{2+}$ (0.3 mM) in deoxygenated aqueous solution, recorded in the absence (black dots) and presence (red dots) of allomelanin NPs.

Since molecular oxygen, in its electronic ground state, is a triplet and is known to quench triplet excited states, we further investigated whether oxygen could quench the excited state of allomelanin. The nanosecond-to-microsecond decay of allomelanin NPs upon 355 nm excitation was recorded under both oxygenated (ambient air) and deoxygenated (Ar-bubbled for 30 minutes) conditions. As shown in Figure S26, no differences were observed in the decay profiles, confirming that allomelanin excited states are not significantly quenched by molecular oxygen.

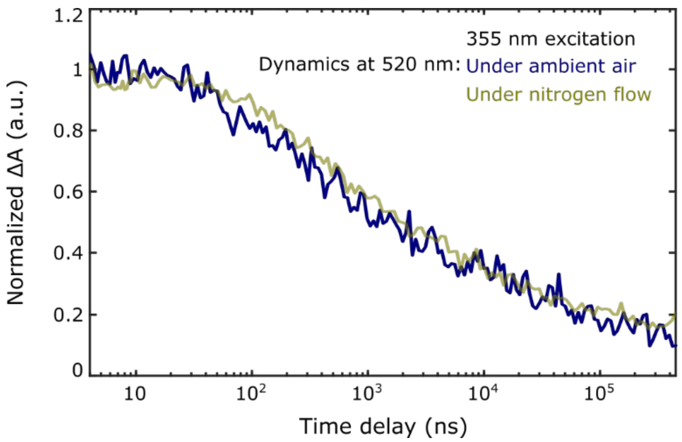

Figure S26: Ns-TA kinetics of allomelanin NPs upon 355 nm excitation, recorded in the nanosecond-to-microsecond time range under oxygenated and deoxygenated conditions.

Finally, to verify the absence of singlet oxygen formation—a hallmark of triplet energy transfer to molecular oxygen—we analyzed the NIR emission (1270 nm) of an oxygenated allomelanin NP suspension upon excitation. As shown in Figure S27, no detectable singlet oxygen emission was observed, using the well-known C_{60} sensitizer as a reference. This confirms that allomelanin does not exhibit measurable triplet-state-mediated energy transfer to molecular oxygen under these conditions.

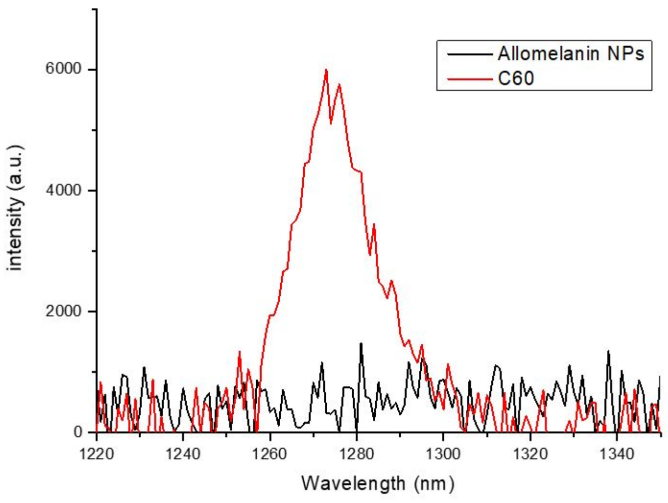

Figure S27: NIR emission spectrum of an oxygenated aqueous suspension of allomelanin NPs, compared to C_{60}. The wavelength range focuses on the 1270 nm emission, characteristic of singlet oxygen.

8. Comparison of allomelanin's and eumelanin's key features

Feature	Allomelanin (This Work)	Eumelanin
**Main Precursor used**[13]	**Nitrogen-free** (1,8-DHN)	**Nitrogen-rich** (DHI or DHICA)
**Chemical/Structural Heterogeneity**[13,14]	**Moderate to High:** Multiple chemical species form after oxidation; simpler precursors suggest potentially lower heterogeneity with respect to eumelanin	**High:** Complex mixture of oxidation states, diverse monomers, and aggregation patterns
**Origin of Broad Absorption Spectrum**	**Excitonic Model:** Strongly coupled chromophores within homo- and hetero-aggregates of monomers, spreading the excitonic couplings from the UV to the visible energies	**Charge Transfer (CT) + Excitonic States:**[2,3] Requires electron donor-acceptor sub-populations possessing mixed CT and excitonic states
**Wavelength-Dependent Transient Absorption**	**Low:** No hole-burning observed; similar TA spectra for different excitation wavelengths; UV and visible excitation lifetimes are very similar	**High:**[2,5–7] Hole-burning effects due to distinct donor-acceptor sub-populations present; accelerated kinetics from UV to visible excitation
**Main Excited-State Species**	**SQR-like Species:** Formed with a 24 ps lifetime, stabilized through electron and proton transfer reactions	**CT States:**[2,6,15–17] Form within ~50 fs; exact molecular origin still under study; speculated to form within hydroquinone/quinone pairs
**Long-Lived Transient Signals**	**High amplitude:** Populations persist up to 450 μs, correlating with EPR signals	**Low amplitude:**[2,16] Main decay within 10 ps; low residual signals persist longer (> 1ns) and have been associated to radical quantum yields
**Antioxidant activity**[10,14,18]	**Higher**	**Lower**
**Light-dependent EPR signals**[19,20]	**Reversible Redox Chemistry:** Linked to hydroquinone- and quinone- like ↔ SQR-like equilibrium, dictated by electron and proton transfer reactions	
**Light-enhanced antioxidant activity**	**Yes:** SQR-like species enhance radical degradation (DPPH assay used)	**Unclear:**[19,21] Potential photoprotective or photodamaging roles have been reported

Table S7: A comparative summary of key features observed in melanins, highlighting distinctive properties between allomelanin NPs studied in this work and eumelanins reported in the literature.

9. Light-induced comproportionation equilibrium schemes

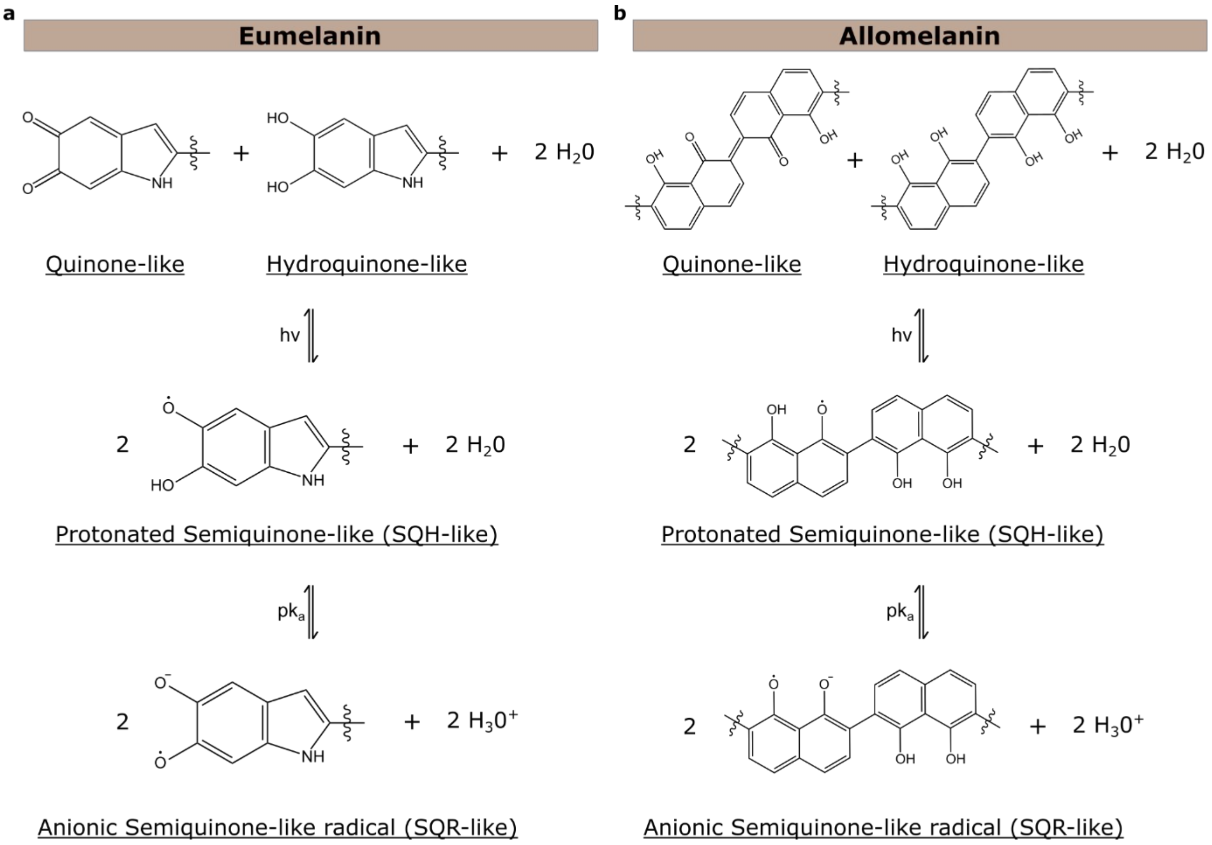

Figure S28: Proposed pathway for light-activated semiquinone-like radical formation (light-induced comproportionation) in (a) eumelanins[19,20] and (b) allomelanins, explaining the light-dependent EPR signals. Upon illumination, a one-electron redox reaction transfers an electron and a proton from a hydroquinone-like to a quinone-like structure, forming protonated semiquinone-like (SQH-like) intermediates. The system's pKa subsequently dictates the generation of the anionic semiquinone-like radical (SQR-like), which predominates under the experimental conditions used in this study (aqueous environment, pH 7). When the light source is removed, the transient photoproducts decay, and the redox equilibrium reverts to its original state. It is important to emphasize that the structure of melanins remains complex and not fully determined. The depicted chemical structures are representative, reflecting the most abundant molecular species found in these melanins. The redox equilibrium between hydroquinone- and quinone-like structures shown here can be generalized to other similar pairs within the allomelanin framework.

10. Proposed photochemical pathways in allomelanin

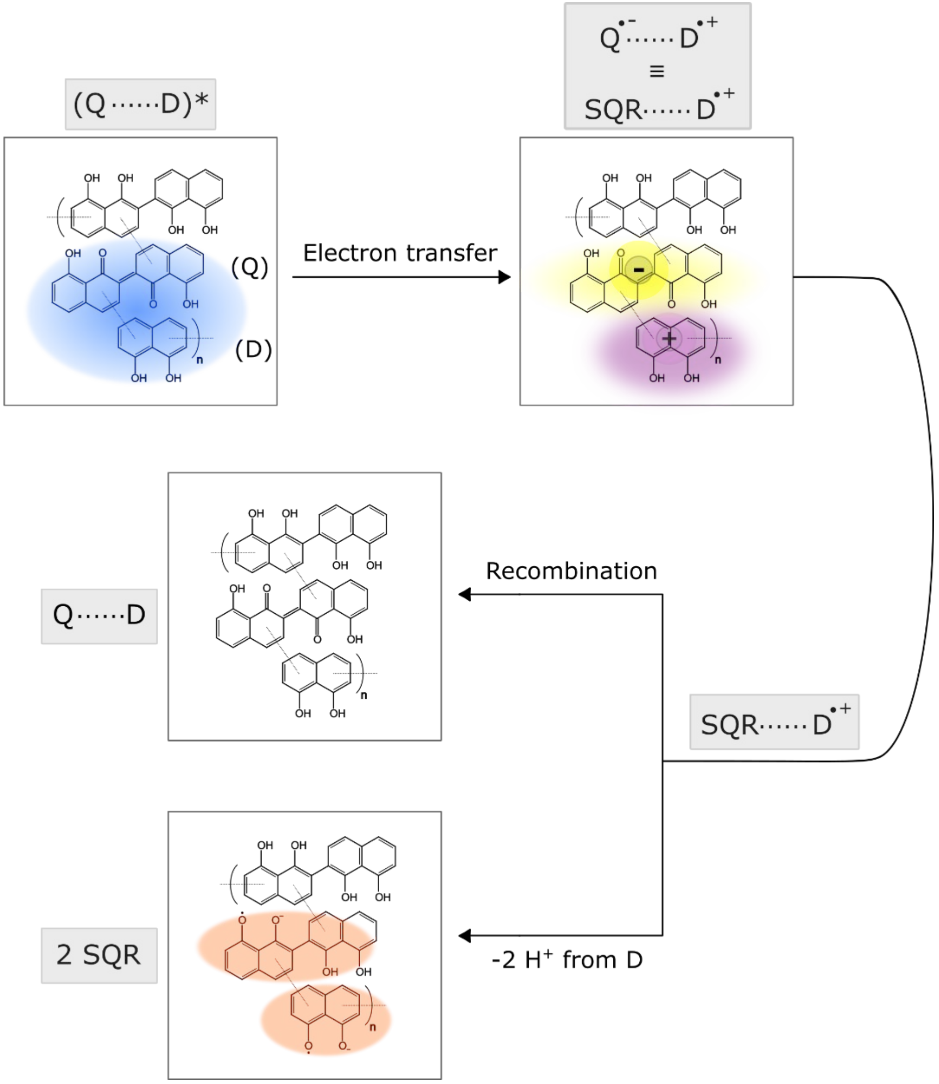

Figure S29: Proposed photochemical pathways following the photoexcitation of allomelanin. Electron transfer is favorable between closely packed quinone-like (Q) and dihydroquinone-like (D) pairs. The quinone-like radical anion formed during this process is equivalent to a semiquinone-like radical anionic structure (SQR). The resulting charge-transfer state, consisting of SQR-like species and D-like structures, can either recombine or undergo further stabilization through the loss of two protons, forming stabilized SQR species. It is important to note that the structure of allomelanin remains complex and undetermined. The depicted structures represent a simplified schematic designed to help the reader visualize potential interactions between closely packed Q-D pairs after photoexcitation. These processes can be generalized to other Q-D pairs within the allomelanin framework.

While the precise origins of the early states contributing to EAS_1 and EAS_2 in our global fitting approaches are beyond the main scope of this study, theoretical and experimental evidence strongly suggests that the 24 ps lifetime (EAS_3) corresponds to stabilized SQR-like species. Notably, no spectral evolution is observed from 24 ps to 450 μs, indicating minimal changes in the excited-state character during this period. For completeness, we tried to provide insight into the origin of the initial states, proposing a mixing of excitonic and CT states. Fluence-dependent measurements and spectral features further support this hypothesis, with initially excitonic character being predominant, and with the downhill relaxation enhancing the CT character, which eventually evolves into a stabilized CT state within 24 ps.

Overall, as stated in detail in the main text, the photophysical and photochemical pathways of allomelanin involve excited-state CT interactions between closely Q-D-like pairs. While not all photoexcitations result in Q-D interactions, excitons in melanins are short-lived, typically undergoing internal conversion or phototransformation into CT states within tens of picoseconds.[2,16]

Our proposed photochemical pathway (Figure S29) entails electron and proton transfer reactions between Q-D pairs during the 24 ps lifetime, accompanied by the decay of any excitonic contribution present, resulting in the final photoproducts of bound CT states (SQR-D^+) and stabilized SQR species. Additionally, proton loss from D^+ species, facilitated by the surrounding water network and redox-active groups in allomelanin, can further stabilize the SQR species. Based on our hypothesis, the transient datasets determining the excited state from 24 ps to 450 μs, can mainly originate from SQR-like excitations. This mechanistic pathway proposed, is further supported by the theoretical simulations performed for the SQR-like fundamental unit of 1,8-DHN derived allomelanin, the 1,8-napthoquinone radical anion (see below).

An alternative photochemical pathway following the excited-state interaction of closely packed Q-D pairs could involve direct proton-coupled electron transfer (PCET), resulting in the formation of SQH-like species.[19,20] However, under our experimental conditions, this pathway would likely lead to the subsequent formation of SQR species, as SQH is unstable and rapidly converts into the predominant SQR species in water at pH 7.[19,20] Furthermore, no PA peaks at 300 nm and 700 nm,[11] which would confirm the presence of SQH-like species as intermediates, are observed. These findings strongly support a stepwise electron and proton transfer mechanism, in which SQR like species are predominant species over the time span of the reaction, as illustrated in Figure S29.

11. Estimation of radical quantum yields through TA measurements

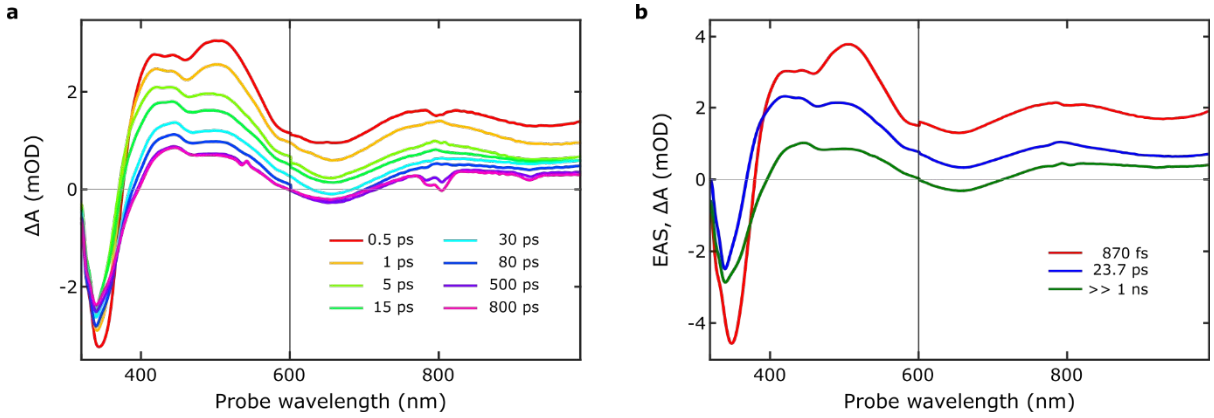

Figure S30: (a) Fs-TA spectra of allomelanin NPs in water at selected pump-probe delay times up to 1 ns following 266 nm excitation. The probe range spans 320–980 nm, achieved by merging two consecutive measurements divided at the 600 nm probe line. **(b)** EAS components and their corresponding time constants, obtained through global fitting of the displayed datasets.

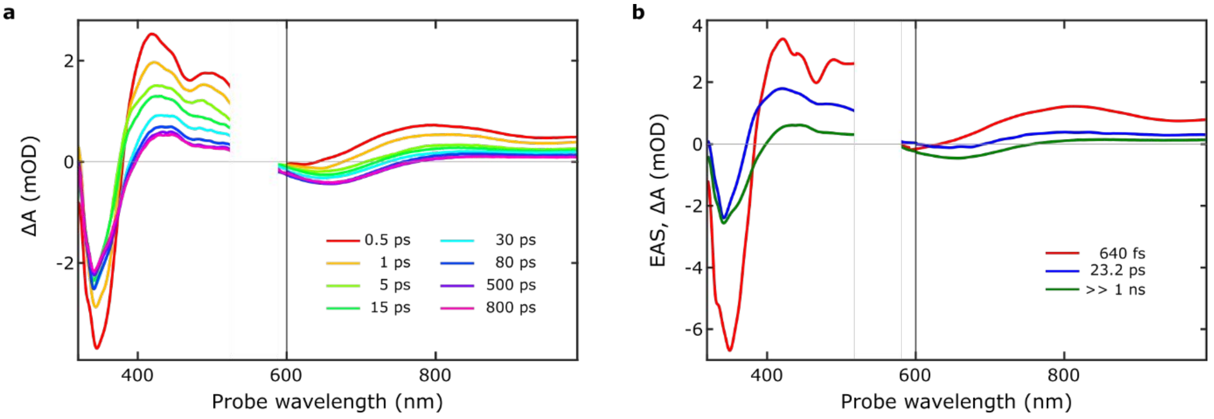

Figure S31: (a) Fs-TA spectra of allomelanin NPs in water at selected pump-probe delay times up to 1 ns following 550 nm excitation. The probe range spans 320–980 nm, obtained by merging two consecutive measurements divided at the 600 nm probe line. Regions with strong pump scattering have been omitted. **(b)** EAS components and their corresponding time constants, derived through global fitting of the displayed datasets.

We briefly outline the method used to estimate the absolute and relative quantum yields of the SQR-like species. Based on the excitation fluence used in the experiments shown

in Figures S30 and S31, and the allomelanin population absorbed at the respective wavelengths, the concentration of absorbed photons is estimated to be 6.8×10^{-6} mol L^{-1} for 266 nm excitation and 1.1×10^{-5} mol L^{-1} for 550 nm excitation (Table S8).

At early timescales (before the 24 ps lifetime component), multi-species dynamics and potential mixing of excitonic and charge transfer states are observed, with the equilibrium favoring bound excitonic species (see “Fluence dependent transient absorption measurements” section). Additionally, the presence of multiple species with different molar absorption coefficients and the overlap between GSB and PA signals complicate the assignment of any spectral evolution. As such, using GSB recovery as a guide to determine the recombination population is not a reliable method.

The SQR-like species are proposed to dominate the excited state after the 24 ps lifetime. Consequently, the 800–980 nm probe region in the NIR within EAS_3 offers a background-free window, as GSB is negligible, for accurately quantifying the formed SQR-like species. The SQR-like concentration is estimated from the transient absorption signal in the NIR region of EAS_3, measured at 0.43 mOD and 0.15 mOD for 266 nm and 550 nm excitations, respectively, along with the molar absorption coefficient of the 1,8-naphthoquinone radical anion (1,8-NQRA) species' NIR band. We note that we assign the SQR-like species to mainly localized 1,8-NQRA excitations in origin.

As such, significant uncertainty arises from using two quantum chemistry methods to calculate the molar absorption coefficient—one based on the work of Manini et al.[11] and the other derived from our own calculations—which results in a threefold difference in the estimated absolute quantum yields (Table S8). Nevertheless, we judge that reporting these values is still of high importance, as future experimental determinations of the precise molar absorption coefficient for 1,8-NQRA may refine the absolute quantum yield calculations.

Given the current datasets, we emphasize the relative quantum yield between 266 nm and 550 nm excitations, as this measurement remains unaffected by uncertainties in the molar absorption coefficient (Table S8). The relative quantum yield is four times higher under UV excitation (266 nm) compared to visible excitation (550 nm).

Feature	Excitation at 266 nm	Excitation at 550 nm
Concentration of absorbed photons (mol L^{-1})	(6.8±1) x 10^{-6}	(1.1±0.2) x 10^{-5}
Transient signal of the SQR-like species in the NIR [a] (mOD)	0.43±0.01	0.15±0.01
Molar absorption coefficient of 1,8-NQRA in the NIR (M^{-1} cm^{-1})	2280 [b]	
Molar absorption coefficient of 1,8-NQRA in the NIR (M^{-1} cm^{-1})	725 [c]	
Absolute quantum yield of SQR-like species formed (%)	28±4 [d]	7±1 [d]
Absolute quantum yield of SQR-like species formed (%)	87±13 [e]	21±4 [e]
Relative quantum yield of SQR-like species formed (266 nm yield / 550 nm yield)	~ 4	

[a] Monitoring the average transient absorption signal on the 800-980 nm probe region in the NIR in EAS_3. The peak is assigned to the photoinduced concentration of the SQR-like species.

[b] The average theoretical value corresponding to the NIR peak of the 1,8-NQRA, as reported by Manini et. al.[11]

[c] The average theoretical value corresponding to the NIR peak of the 1,8-NQRA estimated by our work

[d] Calculated using the molar absorption coefficient of 2280 M^{-1} cm^{-1}

[e] Calculated using the molar absorption coefficient of 725 M^{-1} cm^{-1}

Table S8: Experimental values of absorbed photon concentrations for allomelanin NPs dispersed in water, corresponding to the experiments shown in Figures S30 and S31 for excitations at 266 nm and 550 nm. The concentration of formed SQR-like species is estimated from the weighted average of the TA signal in the 800–980 nm probe region in the NIR, as shown in EAS_3, and the molar absorption coefficient of the 1,8-NQRA NIR band. The molar absorption coefficient is derived using two distinct quantum chemistry methods, resulting in different absolute quantum yields for the formed SQR-like species. Nevertheless, the relative radical quantum yield is four times higher under UV excitation at 266 nm compared to visible excitation at 550 nm.

12. Simulations on fundamental allomelanin units

Computation method

The 1,8-naphthoquinone radical anion structure was optimized at the DFT level of theory using the PBE0 functional and the aug-cc-pVDZ basis set. The water environment was treated implicitly via a PCM model.

The theoretical absorption spectrum of the 1,8-naphthoquinone radical anion was then calculated by an electrostatic embedding QM/MM scheme with the following system partitioning: (i) high layer, consisting of the chromophore structure, treated at the QM level; (ii) low layer, containing 2000 solvent molecules (water), treated at the MM level. The low layer was equilibrated around the QM region by classical MD simulation, where the electrostatic potential of the chromophore was modelled by ESP charges fitted from the DFT electron density. 100 uncorrelated solvent snapshots were then taken from the classical MD by extracting the solvent configuration every 2 ps of MD.

The vertical excitation energies and transition dipole moments were evaluated at XMS-RASPT2 level with a state average of 8. In particular, a 17-electron active space was designed by placing 6 π-type orbitals along with 2 oxygen n-type orbitals in RAS1 and 6 π^* -type orbitals in RAS3. Four holes and four electrons were allowed in RAS1 and RAS3, respectively. The molecular orbitals included in the active space and their partitioning are also shown in Figure S32. All CASPT2 calculations were performed with zero IPEA shift and an imaginary shift of 0.2.

The transition dipole moment between the doublet radical states was calculated using the RASSI routine, while the NTO analysis (Figure S33) was performed using the WFA routine of Molcas[22].

All quantum chemical calculations described here were performed with the cc-pVDZ basis set, and relying on the Cholesky decomposition[23] to speed up the evaluation of the electron integrals. The absorption spectrum was obtained by a Gaussian convolution of the transition energy and the transition dipole moment with a standard deviation of 0.08 eV. The final spectra shown in Figure 6c of the main text are the results of averaging over the 100 uncorrelated solvent configurations.

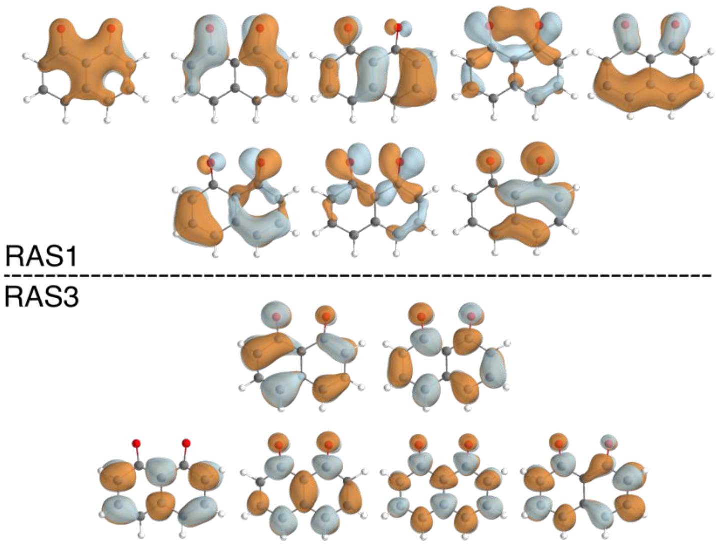

Figure S32: Active space employed in RASPT2 calculations of 1,8-naphthoquinone radical anion.

Natural transition orbital analysis

Transition	α-Hole	α-Particle		β-Hole	β-Particle		E [eV]	OS [a.u.]
D_1-D_2				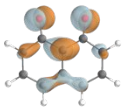	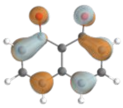	(0.64)	1.27	0.036
D_1-D_3				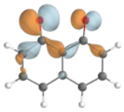	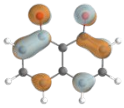	(0.63)	1.95	0.008
D_1-D_4	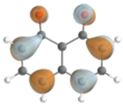	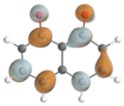	(0.52)	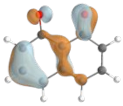	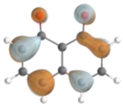	(0.10)	2.51	0.016
D_1-D_5	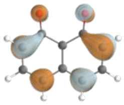	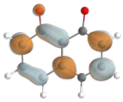	(0.27)	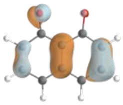	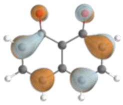	(0.40)	2.66	0.006
D_1-D_6				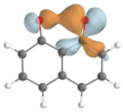	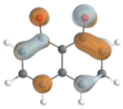	(0.56)	3.32	0.007
D_1-D_7	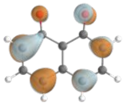	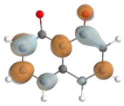	(0.23)	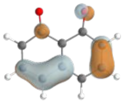	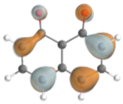	(0.24)	3.67	0.062
D_1-D_8	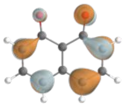	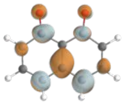	(0.27)	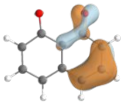	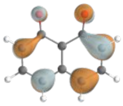	(0.24)	3.77	0.011

Figure S33: Natural transition orbital analysis for the seven doublet to doublet transitions giving rise to the 1,8-naphthoquinone radical anion absorption spectrum. Both the α and β NTOs are here reported with the corresponding participation weights to the total excited state wave function. These are evaluated considering only the α part of the ground state doublet. The here presented data are extracted from one of the uncorrelated 100 solvent snapshoots.

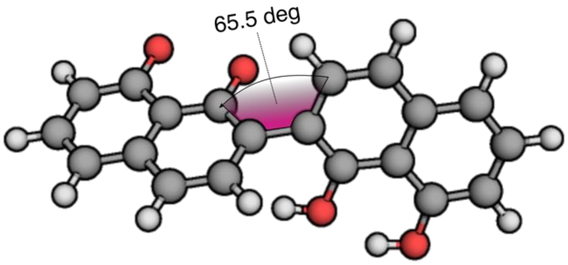

Figure S34: Example of a possible SQR-like dimeric structure, based on the most abundant 2-2′ dimers in 1,8-DHN-derived allomelanin. The optimized ground-state structure was obtained through DFT optimization using the PBE0 functional and the aug-cc-pVDZ basis set. The water environment was treated implicitly using a PCM model. The dihedral angle between the two naphthalene units is highlighted with a shaded area.

13. References

(1) Mavridi-Printezi, A.; Giordani, S.; Menichetti, A.; Mordini, D.; Zattoni, A.; Roda, B.; Ferrazzano, L.; Reschiglian, P.; Marassi, V.; Montalti, M. The Dual Nature of Biomimetic Melanin. *Nanoscale* **2024**, *16* (1), 299–308. https://doi.org/10.1039/D3NR04696F.

(2) Petropoulos, V.; Mavridi-Printezi, A.; Menichetti, A.; Mordini, D.; Kabacinski, P.; C. Gianneschi, N.; Montalti, M.; Maiuri, M.; Cerullo, G. Sub-50 Fs Formation of Charge Transfer States Rules the Fate of Photoexcitations in Eumelanin-Like Materials. *J Phys Chem Lett* **2024**, *15* (13), 3639–3645. https://doi.org/10.1021/acs.jpclett.4c00170.

(3) Mavridi-Printezi, A.; Menichetti, A.; Ferrazzano, L.; Montalti, M. Reversible Supramolecular Noncovalent Self-Assembly Determines the Optical Properties and the Formation of Melanin-like Nanoparticles. *J Phys Chem Lett* **2022**, *13* (42), 9829–9833. https://doi.org/10.1021/acs.jpclett.2c02239.

(4) Ju, K.-Y.; Fischer, M. C.; Warren, W. S. Understanding the Role of Aggregation in the Broad Absorption Bands of Eumelanin. *ACS Nano* **2018**, *12* (12), 12050–12061. https://doi.org/10.1021/acsnano.8b04905.

(5) Thompson, A.; Robles, F. E.; Wilson, J. W.; Deb, S.; Calderbank, R.; Warren, W. S. Dual-Wavelength Pump-Probe Microscopy Analysis of Melanin Composition. *Sci Rep* **2016**, *6* (1), 36871. https://doi.org/10.1038/srep36871.

(6) Kohl, F. R.; Grieco, C.; Kohler, B. Ultrafast Spectral Hole Burning Reveals the Distinct Chromophores in Eumelanin and Their Common Photoresponse. *Chem Sci* **2020**, *11* (5), 1248–1259. https://doi.org/10.1039/C9SC04527A.

(7) Ilina, A.; Thorn, K. E.; Hume, P. A.; Wagner, I.; Tamming, R. R.; Sutton, J. J.; Gordon, K. C.; Andreassend, S. K.; Chen, K.; Hodgkiss, J. M. The Photoprotection Mechanism in the Black–Brown Pigment Eumelanin. *Proceedings of the National Academy of Sciences* **2022**, *119* (43). https://doi.org/10.1073/pnas.2212343119.

(8) Chandrabose, S.; Chen, K.; Barker, A. J.; Sutton, J. J.; Prasad, S. K. K.; Zhu, J.; Zhou, J.; Gordon, K. C.; Xie, Z.; Zhan, X.; Hodgkiss, J. M. High Exciton Diffusion Coefficients in Fused Ring Electron Acceptor Films. *J Am Chem Soc* **2019**, *141* (17), 6922–6929. https://doi.org/10.1021/jacs.8b12982.

(9) Rehhagen, C.; Rather, S. R.; Schwarz, K. N.; Scholes, G. D.; Lochbrunner, S. Comparison of Frenkel and Excimer Exciton Diffusion in Perylene Bisimide Nanoparticles. *J Phys Chem Lett* **2023**, *14* (19), 4490–4496. https://doi.org/10.1021/acs.jpclett.3c00364.

(10) Zhou, X.; McCallum, N. C.; Hu, Z.; Cao, W.; Gnanasekaran, K.; Feng, Y.; Stoddart, J. F.; Wang, Z.; Gianneschi, N. C. Artificial Allomelanin Nanoparticles. *ACS Nano* **2019**, *13* (10), 10980–10990. https://doi.org/10.1021/acsnano.9b02160.

(11) Manini, P.; Bietti, M.; Galeotti, M.; Salamone, M.; Lanzalunga, O.; Cecchini, M. M.; Reale, S.; Crescenzi, O.; Napolitano, A.; De Angelis, F.; Barone, V.; d'Ischia, M. Characterization and Fate of Hydrogen-Bonded Free-Radical Intermediates and Their

Coupling Products from the Hydrogen Atom Transfer Agent 1,8-Naphthalenediol. *ACS Omega* **2018**, *3* (4), 3918–3927. https://doi.org/10.1021/acsomega.8b00155.

(12) Pukalski, J.; Mokrzyński, K.; Chyc, M.; Potrzebowski, M. J.; Makowski, T.; Dulski, M.; Latowski, D. Synthesis and Characterization of Allomelanin Model from 1,8-Dihydroxynaphthalene Autooxidation. *Sci Rep* **2025**, *15* (1), 567. https://doi.org/10.1038/s41598-024-84405-0.

(13) Cao, W.; Zhou, X.; McCallum, N. C.; Hu, Z.; Ni, Q. Z.; Kapoor, U.; Heil, C. M.; Cay, K. S.; Zand, T.; Mantanona, A. J.; Jayaraman, A.; Dhinojwala, A.; Deheyn, D. D.; Shawkey, M. D.; Burkart, M. D.; Rinehart, J. D.; Gianneschi, N. C. Unraveling the Structure and Function of Melanin through Synthesis. *J Am Chem Soc* **2021**, *143* (7), 2622–2637. https://doi.org/10.1021/jacs.0c12322.

(14) Mavridi-Printezi, A.; Mollica, F.; Lucernati, R.; Montalti, M.; Amorati, R. Insight into the Antioxidant Activity of 1,8-Dihydroxynaphthalene Allomelanin Nanoparticles. *Antioxidants* **2023**, *12* (8), 1511. https://doi.org/10.3390/antiox12081511.

(15) Grieco, C.; Kohl, F. R.; Hanes, A. T.; Kohler, B. Probing the Heterogeneous Structure of Eumelanin Using Ultrafast Vibrational Fingerprinting. *Nat Commun* **2020**, *11* (1), 4569. https://doi.org/10.1038/s41467-020-18393-w.

(16) Grieco, C.; Kohl, F. R.; Kohler, B. Ultrafast Radical Photogeneration Pathways in Eumelanin †. *Photochem Photobiol* **2023**, *99* (2), 680–692. https://doi.org/10.1111/php.13731.

(17) Matta, M.; Pezzella, A.; Troisi, A. Relation between Local Structure, Electric Dipole, and Charge Carrier Dynamics in DHICA Melanin: A Model for Biocompatible Semiconductors. *J Phys Chem Lett* **2020**, *11* (3), 1045–1051. https://doi.org/10.1021/acs.jpclett.9b03696.

(18) Lino, V.; Manini, P. Dihydroxynaphthalene-Based Allomelanins: A Source of Inspiration for Innovative Technological Materials. *ACS Omega* **2022**, *7* (18), 15308–15314. https://doi.org/10.1021/acsomega.2c00641.

(19) Mostert, A. B.; Rienecker, S. B.; Noble, C.; Hanson, G. R.; Meredith, P. The Photoreactive Free Radical in Eumelanin. *Sci Adv* **2018**, *4* (3). https://doi.org/10.1126/sciadv.aaq1293.

(20) Bailey, C. G.; Nothling, M. D.; Fillbrook, L. L.; Vo, Y.; Beves, J. E.; McCamey, D. R.; Stenzel, M. H. Polydopamine as a Visible-Light Photosensitiser for Photoinitiated Polymerisation. *Angewandte Chemie International Edition* **2023**, *62* (20). https://doi.org/10.1002/anie.202301678.

(21) Solano, F. Photoprotection *versus* Photodamage: Updating an Old but Still Unsolved Controversy about Melanin. *Polym Int* **2016**, *65* (11), 1276–1287. https://doi.org/10.1002/pi.5117.

(22) Plasser, F.; Krylov, A. I.; Dreuw, A. Libwfa: Wavefunction Analysis Tools for Excited and Open-shell Electronic States. *WIREs Computational Molecular Science* **2022**, *12* (4). https://doi.org/10.1002/wcms.1595.

(23) Pedersen, T. B.; Aquilante, F.; Lindh, R. Density Fitting with Auxiliary Basis Sets from Cholesky Decompositions. *Theor Chem Acc* **2009**, *124* (1–2), 1–10. https://doi.org/10.1007/s00214-009-0608-y.
